# Diet, Food, and Nutritional Exposures and Inflammatory Bowel Disease or Progression of Disease: an Umbrella Review

**DOI:** 10.1016/j.advnut.2024.100219

**Published:** 2024-04-08

**Authors:** Camilla Christensen, Andrea Knudsen, Erik K Arnesen, Jan Gunnar Hatlebakk, Ida Sofie Sletten, Lars T Fadnes

**Affiliations:** 1 Department of Global Public Health and Primary Care, University of Bergen, Bergen, Norway; 2 Department of Nutrition, Institute of Basic Medical Sciences, University of Oslo, Oslo, Norway; 3 Department of Clinical Medicine, University of Bergen, Bergen, Norway; 4 Norwegian Centre of Competence in Functional Gastrointestinal Disorders, Haukeland University Hospital, Bergen, Norway; 5 The Medical Library, University of Bergen, Bergen, Norway; 6 Department of Addiction Medicine, Haukeland University Hospital, Bergen, Norway

**Keywords:** dietary patterns, food groups, inflammatory bowel disease, Crohn’s disease, ulcerative colitis

## Abstract

Inflammatory bowel disease (IBD), including Crohn’s disease (CD) and ulcerative colitis (UC), contributes to substantial morbidity. Understanding the intricate interplay between dietary factors and the incidence and progression of IBD is essential for developing effective preventative and therapeutic strategies. This umbrella review comprehensively synthesizes evidence from systematic reviews and meta-analyses to evaluate these complex associations. Dietary factors associated with an increased incidence and/or progression of IBD include a high intake of red and processed meat, other processed foods, and refined sugars, together with a low intake of vegetables, fruits, and fiber. For most other food groups, the results are mixed or indicate no clear associations with IBD, CD, and UC. Some differences seem to exist between UC and CD and their risk factors, with increased intake of dietary fiber being inversely associated with CD incidence but not clearly associated with UC. Dietary fiber may contribute to maintaining the gut epithelial barrier and reduce inflammation, often through interactions with the gut microbiota. This seems to play an important role in inflammatory mechanisms in the gut and in IBD incidence and progression. Diets low in fermentable saccharides and polyols can alleviate symptom burden, but there are concerns regarding their impact on the gut microbiota and their nutritional adequacy. Mediterranean diets, vegetarian diets, and a diet low in grains, sugars, and lactose (specific carbohydrate diet) are also associated with lower incidence and/or progression of IBD. The associations of dietary patterns are mirrored by inflammatory biomarkers. IBD is typically treated pharmaceutically; however, many patients have a suboptimal response to medical treatments. The findings from this umbrella review could provide evidence for nutritional counseling and be a valuable addition to traditional treatment plans for IBD.

This systematic review was registered at PROSPERO as CRD440252.

**Statement of Significance**

Many studies have presented associations between diet and inflammatory bowel disease, but a comprehensive and up-to-date overview is lacking. We present a range of strong associations between intake of food groups and incidence of inflammatory bowel disease as well as progression of disease. This could enable the provision of dietary guidance as part of the management of inflammatory bowel disease.

## Introduction

Inflammatory bowel disease (IBD) is on the rise worldwide, with ∼7 million people currently having the diagnosis, taking a toll on patients’ health as well as being a growing economic burden to society [1]. In 2017, the loss of nearly 2 million disability-adjusted life years was attributed to IBD [1]. Thus, to prevent a further increase, knowledge of risk factors for both disease development and progression is fundamental.

IBD includes Crohn’s disease (CD) and ulcerative colitis (UC), but also less common diseases such as microscopic colitis. IBD is characterized by inflammation of the gastrointestinal tract and dysregulation of immune responses, typically with relapses and remissions [2,3]. Long-term use of immunosuppressive, anti-inflammatory, biological, and immunomodulatory medications are often required for management of the disease [4,5], whereas dietary interventions have traditionally been less emphasized.

In the past decade, substantial progress has been made in unraveling the pathogenesis of IBD [3]. IBD is now understood to comprise complex interactions between genetics, microbial, and environmental factors that lead to dysregulation of the mucosal immune system [2,3]. An important hypothesis is that the increase in incidence and prevalence of IBD is associated with a modern lifestyle and diet [6]. Several different dietary exposures have been investigated for their associations with IBD, CD, and UC incidence and progression [[7], [8], [9], [10], [11], [12], [13], [14], [15], [16], [17]], but a comprehensive and up-to-date overview is lacking. One umbrella review on environmental risk factors and IBD was conducted in 2019 that did include some dietary factors [18]. However, the review did not cover the full extent of dietary exposures, and many studies have been published since then.

The aim of this umbrella review was to present and evaluate all the systematic reviews and meta-analyses on diet and its association with incidence of IBD, UC, and CD, as well as progression and remission of the diseases.

## Methods

We conducted an umbrella review to summarize evidence from systematic reviews and meta-analyses on how diet, food, and nutritional exposures are associated with risk of IBD or progression of the disease. The protocol was registered in PROSPERO as CRD440252.

### Literature search

**FIGURE 1:**
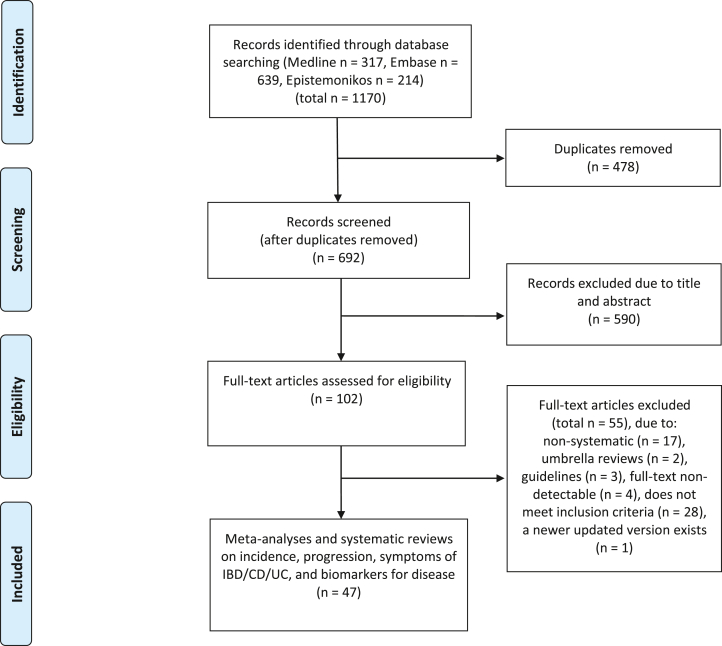
Study selection for the umbrella analysis of different dietary exposures and associations with inflammatory bowel disease. CD, Crohn’s disease; IBD, inflammatory bowel disease; UC, ulcerative colitis.

A comprehensive literature search of Medline Ovid, Embase Ovid, and Epistemonikos was carried out on 14 June, 2023. The search combined relevant synonyms and subject headings using Boolean operators with search terms inflammatory bowel disease, UC or Morbus Crohn, diet, food or nutrition, and systematic reviews or meta-analyses. We retrieved 1170 records and removed 478 duplicates, with 692 articles remaining (Figure 1). We adhered to the PRISMA guidelines for this umbrella review [19]. The literature search was conducted with the assistance of an academic librarian and peer-reviewed by another academic librarian. The complete search strategy can be found in Supplementary Text S1.

### Eligibility criteria and types of studies

We systematically searched the databases for systematic reviews and meta-analyses of observational studies and randomized controlled trials that investigated associations between dietary patterns, individual food items, and nutritional exposures and the incidence and progression of inflammatory bowel disease. Inclusion and exclusion criteria are described below. We had no restrictions on publication date. Articles written in languages other than English, Norwegian, Danish, Swedish, or German were excluded.

**Inclusion criteria:**

• Study types: systematic reviews and meta-analyses of observational studies with follow-up of patients and randomized controlled trials (all types).
• Exposure: diet, food, and nutritional exposures.
• Comparators: high compared with low consumption, nutritional exposures compared with control diet, per unit (e.g., grams).
• Outcome: incidence and progression of IBD and subtypes (see details below).
• Publication status: articles indexed in Medline, Embase, and Epistemonikos.

**Exclusion criteria:** We excluded animal studies and studies only providing region-specific estimates, sources without full text articles (such as conference abstracts), and studies exclusively investigating dietary supplements.

### Types of outcome measures

The outcome measures included were IBD including CD, UC, microscopic colitis, diversion colitis, Behçet’s disease, early onset IBD, and indeterminate colitis. Furthermore, disease progression of IBD, including extent of affected intestines or extraintestinal manifestations; regression and risk of complications such as transmural inflammation, stenosis, fistulas, abscess, or perforation; number of symptoms; and need for pharmaceutical, surgical, or endoscopic interventions. We also evaluated different biomarkers for disease, such as fecal calprotectin and C-reactive protein. Outcome measures are presented in Supplementary Tables S1–S3.

### Study selection

After removal of duplicates, the search results were screened in accordance with the inclusion and exclusion criteria by 2 independent reviewers. Conflicts were discussed with the coauthors and resolved by consensus. Rayyan (https://www.rayyan.ai) was used as a screening tool, which facilitates blinded screening by multiple independent reviewers [20,21] (i.e., reviewers going through all records individually and then resolving all conflicts after the blinding is turned off). Rayyan does provide an artificial intelligence function that offers a star rating of articles based on previous screening decisions, but we did not use this. When systematic reviews were overlapping with original articles, the most recent one was favored.

### Data collection process and data items

Data extracted included variables such as time of search, authors and title, study population, number of participants, number of cases (IBD or disease subcategories), study design, IBD incidence/prevalence/disease progression or disease subtype, effect sizes (risk ratio, hazard ratio, absolute risk differences, differences in levels, and 95% confidence intervals [CIs]), heterogeneity, and grading of quality of evidence when available, using Microsoft Excel sheets for data extraction. All extracted data was checked for accuracy by a second reviewer. The number of full text articles assessed was 102, with a total of 47 articles included in the results.

### Risk of bias in individual studies and across studies

Quality assessment of systematic reviews was performed with a modified version of the AMSTAR-2 tool [22]. This was checked by a second reviewer, and disagreements were resolved by consensus. In accordance with this tool, the quality of the reviews was categorized as high/moderate/low/very low (Supplementary Table S4).

### Analysis

**FIGURE 2:**
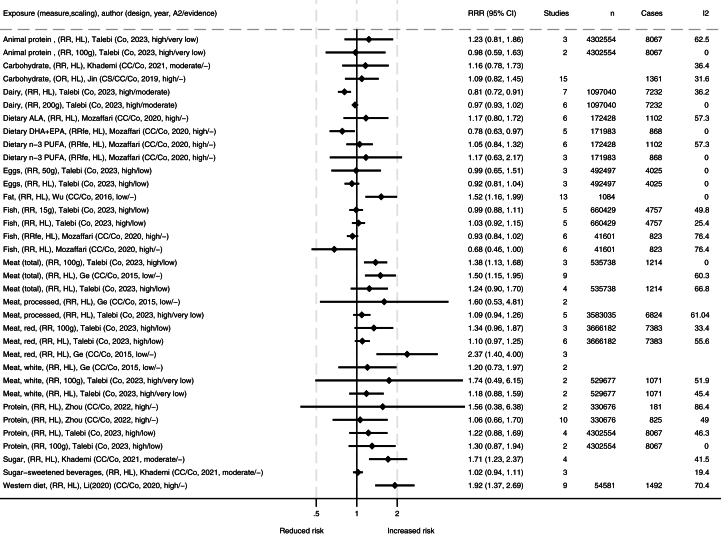
Summary of associations from the meta-analyses between dietary exposures and incidence of inflammatory bowel disease. Publishing year is listed within the parentheses. ∗Comparisons are mostly high compared with low consumption [HL] or presented by grams per day (e.g., 50 g, 100 g, or 200 g), and mostly presented as relative risk ratio (RRR). A2, AMSTAR-2 rating (classified as high/medium/low/critically low); ALA, alpha linolenic acid; CC, case control; CI, confidence interval; Co, cohort; CS, cross sectional; DHA, docosahexaenoic acid; EPA, eicosapentaenoic acid; fe, fixed effects; *I*^2^, heterogeneity (%); MUFA, monounsaturated fatty acid; OR, odds ratio; PUFA, polyunsaturated fatty acid; SFA, saturated fatty acid.

**FIGURE 3:**
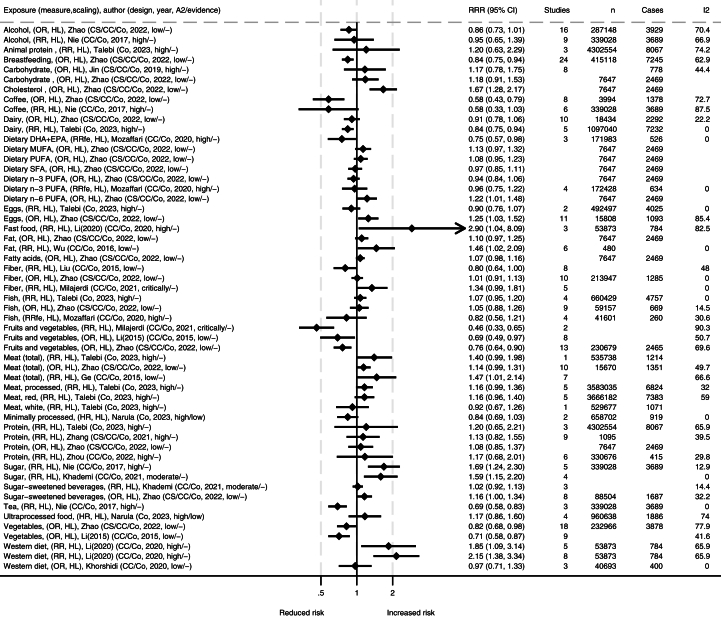
Summary of associations from the meta-analyses between dietary exposures and incidence of ulcerative colitis. Publishing year is listed within the parentheses. ∗Comparisons are mostly high compared with low consumption [HL], and mostly presented as relative risk ratio (RRR). A2, AMSTAR-2 rating (classified as high/medium/low/critically low); CC, case control; Co, cohort; CS, cross sectional; DHA, docosahexaenoic acid; EPA, eicosapentaenoic acid; fe, fixed effects; *I*^2^, heterogeneity (%), MUFA, monounsaturated fatty acid; OR, odds ratio; PUFA, polyunsaturated fatty acid; SFA, saturated fatty acid.

**FIGURE 4:**
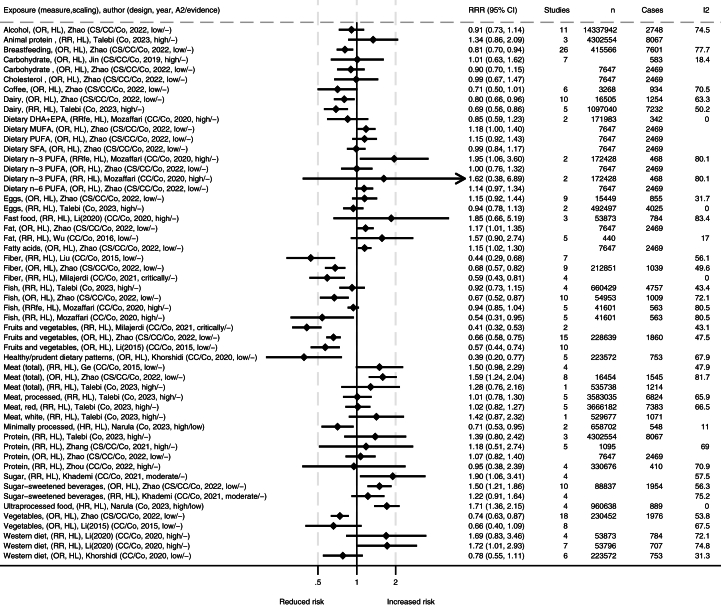
Summary of associations from the meta-analyses between dietary exposures and incidence of Crohn’s disease. Reference number is listed in brackets and search year is listed within the parentheses. ∗Comparisons are mostly high compared with low consumption [HL] and mostly presented as relative risk (RR). A2, AMSTAR-2 rating (classified as high/medium/low/critically low); Co, cohort; CC, case control; CS, xxx; DHA, docosahexaenoic acid; EPA, eicosapentaenoic acid; fe, fixed effects; *I*^2^, heterogeneity (%); MUFA, monounsaturated fatty acid; OR, odds ratio; PUFA, polyunsaturated fatty; SFA, saturated fatty acid.

Tables with extracted data from included studies were made. These data are summarized in FIGURE 2, FIGURE 3, FIGURE 4, visualizing the associations between dietary exposures and IBD-related outcomes for high compared with low (HL), different doses in grams (i.e., change in risk ratio per specified grams/day), or compared with controls. The number of remissions was inverted into nonremissions and presented together with relapses and progressions. We present forest plots for the meta-analyses for each outcome measure. All studies report random effects models unless specified otherwise. The forest plots include information on source/reference, the number of studies, participants, cases, and heterogeneity. We present figures for IBD, UC, and CD, both for incidence and for progression and relapse. We also reanalyzed primary studies presented in the meta-analyses presenting associations with different meat and meat products using relative risks with 95% CIs as the effect estimate, and random effect models to generate the pooled relative risks for highest compared with lowest intakes. Between-study heterogeneity was assessed by the *I*^2^ statistic and presented as a percentage. Stata SE 18 was used for data analysis and graphical presentation.

## Results

A total of 47 systematic reviews, of which 24 included meta-analyses, provided outcome measures for disease and disease progression (Supplementary Tables S1–S3) [[6], [7], [8], [9], [10], [11], [12], [13], [14], [15], [16], [17],[23], [24], [25], [26], [27], [28], [29], [30], [31], [32], [33], [34], [35], [36], [37], [38], [39], [40], [41], [42], [43], [44], [45], [46], [47], [48], [49], [50], [51], [52], [53], [54], [55], [56], [57]], whereas the remaining systematic reviews provided qualitative data without meta-analyses. The quality score in AMSTAR-2 was high for 29 studies, moderate for 3, low for 10, and critically low for 5 (Supplementary Table S4). All studies report data for adults except 2 reporting for children [40,58].

### Incidence of IBD

Several dietary exposures are associated with incidence of IBD (Figure 2). Among these, Western dietary patterns are associated with development of IBD in adults [6] (Supplementary Table S5), with a similar trend seen in children [40]. An association was also seen with a high intake of fat and IBD [16] and for food high in sugars but not for sugar-sweetened beverages [10]. Meat and meat products were generally associated with increased incidence of IBD [7,50], particularly when assessed by dose response [50]. When stratified by location, both studies found a significant association with incidence of IBD in Europe, whereas the findings in Asia and the United States, respectively, were nonsignificant [7,50]. Reanalysis of the primary studies presented in these meta-analyses also ranged between neutral and significant associations with high degree of heterogeneity (Supplementary Figures S1, S2a–e, S3a–f, and S4a–e). For total animal proteins and subproducts of meat, including poultry and fish, estimates were less certain [7,38,50]. There were no clear associations for dietary n–3 (ω-3) PUFA [38] or between the incidence of IBD and the intake of eggs [50] or α-linolenic acid [38]. A protective effect was observed for dairy in the high compared with low category, but no clear association was found with consumption of 200 g of dairy [50]. Breastfeeding was inversely associated with IBD, CD, and UC incidence [30,57].

### Incidence of UC and CD

There were generally comparable results for both UC and CD separately as for IBD in general, with some differences (FIGURE 3, FIGURE 4). A high intake of fruits and vegetables was strongly associated with reduced incidence of UC and CD overall [12,14,34,57]. However, differences in subgroups were detected by some authors, where findings were not as clear or no association was found in Eastern populations in a high compared with low scaling of vegetable or fruit consumption [12,57]. A high intake of dietary fiber was associated with reduced incidence of CD, but the association with UC was unclear [14,34,48,57]. A high intake of coffee was associated with reduced incidence of UC [39,57], and a similar tendency was observed for CD [57]. Minimally processed foods were also associated with reduced incidence of UC [15]. In contrast, ultraprocessed food was associated with increased incidence of CD [15], but the association with UC was not significant. For Western dietary patterns and fast foods in high compared with low, one meta-analysis reported strong associations with the incidence of both UC and CD [6], whereas another meta-analysis of lower quality grading only reported an association with CD incidence, but not for UC [11]. The latter one did however report high heterogeneity for findings on UC [11]. The first one performed subgroup analysis and found significant associations for European and Australian subgroups and CD and UC, but nonsignificant associations were observed in the North American subgroup [6]. Generally, case control studies and retrospective studies reported stronger associations than cohort studies and prospective studies [6]. The largest meta-analysis on total meat and meat products in a comparison of high and low found associations with increased incidence of CD and a nonsignificant trend for UC [57], which was supported by similar trends in other meta-analyses [7,50]. The correlation was stronger in Western compared with Eastern subgroups. Several of the studies found high heterogeneity but attributed this to different study and population types. When assessing different types of meats separately, no clear associations were observed, but a 100 g/d increment of red meat was found to increase incidence of both IBD subtypes in Europe and the United States [50]. Reanalysis of meat consumption showed significantly increased UC incidence for red and processed meat intake and a nonsignificant increase in CD incidence (Supplementary Figure S1). A high intake of sugars was associated with increased incidence of UC and CD [10,39], with similar tendencies for diets high in fats [16,57]. For both UC or CD, no clear associations were reported for intake levels of alcohol, carbohydrates, cholesterol, eggs, fish, dietary n–3 PUFA, and n–6 PUFA [9,39,50,57].

### Progression of disease of IBD, UC, and CD

A diet high in fiber was strongly associated with reduced progression of disease for CD and increased quality of life [13,33,53] (Supplementary Figures S5–S6 ). Both the Mediterranean diet and the specific carbohydrate diet (low in grains, sugars, and lactose) have been associated with improvement of IBD symptoms [8,27] and of quality of life scores [33]. For the specific carbohydrate diet and clinical remission, there is conflicting evidence [13,27,36]. One study compared the Mediterranean diet with the specific carbohydrate diet, but did not have a standard control diet, which made it difficult to properly assess the results [33]. The research on associations between dietary interventions and progression of UC is sparse on CD, which makes it harder to draw conclusions regarding UC [33]. There was high heterogeneity in some studies, which could be caused by undetected small differences within the same dietary patterns across intervention groups [33]. A semivegetarian diet also showed higher clinical remission rates in patients with active CD in one prospective clinical trial; however, this review was rated critically low with the AMSTAR-2 tool [25]. A diet low in fermentable oligosaccharides, disaccharides, monosaccharides, and polyols (FODMAPs) is associated with improvement of several gastrointestinal symptoms [29,41,55] (Supplementary Figure S7). There were no clear associations seen for partial enteral nutrition, n–3 PUFA, carrageenan-free, dairy elimination, or symptom-guided diet with CD, UC, or IBD [13,23,33].

### Biomarkers and mechanisms

Several dietary patterns showed improvement in inflammatory markers such as C-reactive protein in people with IBD, including vegetarian diets [24,27], semivegetarian diets [24], a Mediterranean diet [51], and a high intake of salmon [25]. Some studies only included European countries, whereas others had studies from several continents, and some allowed biological treatment alongside dietary intervention, whereas others did not. A Mediterranean diet was also linked to lower levels of the IBD biomarker fecal calprotectin; this was measured using different scoring systems and questionnaires, finding that a higher adherence to the diet correlated with a lowering of the mentioned inflammatory markers as well as incidence of IBD [27,51]. Further, switching from canola oil to olive oil was associated with decreased levels of C-reactive protein in patients with UC [24]. Similarly, increasing mango intake was also associated with decreased proinflammatory cytokines [24]. An autoimmune protocol that included elimination of some types of grains, vegetables, eggs, milk, coffee, alcohol, nuts and seeds, refined sugars, additives, and processed foods for 6 wk showed a significant reduction in fecal calprotectin and endoscopic inflammation in patients with CD [24]. No significant decrease in C-reactive protein was found in patients with IBD on a diet low in FODMAPs, exclusion of potential allergens (“IgG-diet”), a diet to reduce food microparticles such as titanium dioxide and silicate, added germinated barley, or hydrothermally processed cereals [24,41]. No significant changes in C-reactive protein in UC were found with an increased intake of salmon or increase in fiber at the expense of refined carbohydrates [24].

## Discussion

This umbrella review synthesizes findings on how different food groups, food patterns, and food items are associated with the development and progression of IBD, including CD and UC. A high intake of fruits and vegetables were both strongly associated with reduced incidence of UC and CD [12,14,34,57]. This adds beneficial associations of fruits, vegetables, and dietary fibers with a range of health outcomes such as cardiovascular disease, cancers, and total mortality [[59], [60], [61]]. Inversely, a Western dietary pattern with high intakes of meat, ultraprocessed food, dietary fats, and refined sugars were generally associated with increased incidence of IBD, UC, and CD [6,7,10,16,39,50]. This corresponds well with associations from intakes of these food groups to a range of other health outcomes including cardiovascular disease, type 2 diabetes, and total mortality [[62], [63], [64]]. Ultraprocessed foods were strongly associated with increased incidence of CD, however, not as clearly with UC [15]. Dietary fibers have been found to be inversely associated with increased CD incidence and disease activity; however, the result on UC is less clear [14,34,48,53,57]. For dairy, the results are mixed with slight inverse associations with IBD incidence and disease activity [49,50,57]; on the other hand, there are observations of increased clinical remission in UC when eliminating dairy [13,33]. Results were also mixed for fish intake [38,50,57]. Several dietary patterns have been associated with clinical remission of IBD, including Mediterranean diets [8,27,51], a specific carbohydrate diet low in grains, sugars, and lactose [13,27,33], and a vegetarian or a semivegetarian diet [24,27]. A low-FODMAP diet is associated with decreased symptom burden [41,55]. There are no clear associations for exposures such as eggs, fish, alcohol, carbohydrates, cholesterol, dietary n–3 PUFA, and n–6 PUFA [9,39,50,57].

Dysbiosis of the gut is often seen in patients with IBD [[65], [66], [67]], which is linked to impaired epithelial barrier function and inflammation and may play a role in the pathogenesis of IBD. It has become increasingly clear that the intestinal microbiome is affected by dietary patterns and plays a role in metabolism, epithelial cell integrity, immune cell development, motility, and prevention of colonization of pathogenic strains [67]. Protective species are known to interact with the immune system in a beneficial way and promote homeostasis, whereas other unfavorable species promote inflammation [65]. Thus, it is likely that some of the associations observed for several food groups are mediated through the intestinal microbiome [68]. It has been indicated that high-fat diets have proinflammatory effects on several organs, by changing the gut microbiota, decreasing diversity, upregulating proinflammatory cytokines, inducing oxidative stress in the colon, and disrupting the intestinal barrier [69]. The Mediterranean diet has also been shown to have beneficial properties on the microbiome with a bacterial profile with more anti-inflammatory and less proinflammatory bacteria [66]. Diets high in fermentable fiber stimulate bacteria to produce short-chain fatty acids, such as butyrate, that is known to have anti-inflammatory properties and promote homeostasis [37,65]. Conversely, metabolites produced during protein fermentation by microflora in the colon, such as ammonia and hydrogen sulfide, may lead to alteration of cell membranes in the intestines and reduced barrier integrity [38]. This could explain associations between meat products and incidence of IBD. However, findings on meat and IBD incidence were stronger in Western populations, but only significant in red meat intake and UC in Eastern populations [7,57]. The most recent meta-analysis only found significantly increased incidence with a 100 g/d increment of red meat consumption and for higher intake of red meat in the European subgroup [50]. These studies did report high heterogeneity in findings regarding meat consumption, which may be due to differences in population genetics, food preparation styles, and reporting, as well as study types and other environmental factors. Our reanalysis also showed a clear trend for increased incidence for both UC and CD and different meat consumptions, although only significant findings for UC incidence and red and processed meat consumption. However, we did not stratify by region in our reanalysis. Diets high in fat have also been suggested to impair the intestinal barrier through several proinflammatory mechanisms, including by changes in the gut microbiota [69]. The microbiome develops early in life, and the protective associations for IBD seen for breastfeeding may also be mediated through the microbiome [58,67,70,71].

Inflammatory markers such as C-reactive protein and fecal calprotectin are often used to track disease activity in those with IBD [24]. Studies on dietary patterns and food groups and their effects on inflammatory biomarkers of disease generally mirrors and triangulates the evidence for the associations with IBD, CD, and UC [24,25,27,51]. Dietary fiber seems to help in maintaining the epithelial barrier of the gut through interactions with the gut microbiota, often stimulating anti-inflammatory pathways, as well as acting as a first line of defense against pathogens [37,65]. An important point here is that there are different types of fiber with different mechanisms, which could again influence the pathogenesis of UC and CD differently. The included studies mainly focused on total fiber or different fiber supplements, which makes it difficult to draw conclusions on this now, but it could be an interesting subject for future research. Further, food groups high in dietary fiber such as legumes, whole grains, fruits, and vegetables also naturally contain a range of bioactive components that may be beneficial for IBD, such as flavonoids and polyphenols [14]. The included meta-analyses mostly investigated fruit and vegetable consumption in a high compared with low scaling; in theory, this should mean a greater amount of these foods in the diet should offer more of the mentioned beneficial and anti-inflammatory substances. Interestingly, findings on fruit and vegetable consumption did not always have an inverse effect on incidence in Eastern subgroups but almost always for Western ones. It is difficult to say why this is, but possible explanations may include differences in the genetics of populations, cooking styles, reporting and study types, and availability of different fruits and vegetables. Antioxidant-rich foods such as curcumin seem to have anti-inflammatory properties that could reduce clinical activity in patients with UC and improve clinical and endoscopic remission in patients with IBD [26,32,45]. Dietary patterns, such as the Mediterranean diet, vegetarian and semivegetarian diets, naturally contain larger amounts of these beneficial food groups, which correlates well with the findings on these dietary patterns [8,25,27,33]. The Mediterranean diet also includes a moderate intake of fish; data from one study with random effect models showed inverse associations between fish intake and incidence of CD, which was supported by one other study [38,57]. Intake of DHA and EPA, 2 types of ω-3 acids found in sea products, was found to have an inverse effect on incidence of UC [38], although other studies did not find supporting claims. Interestingly, data on PUFA intake does not mirror fish intake in the 2 IBD subtypes. Data on inflammatory markers in patients with IBD following a Western or standard American diet seems to be currently lacking but could provide useful information if provided in the future.

Diets low in FODMAPs have been reported to reduce symptoms in patients with IBD [41,55]. FODMAPs are short-chain fermentable carbohydrates that can cause water retention leading to diarrhea, gas, and bloating, symptoms that are often substantial among many patients with IBD [72]. However, the overall impact of low-FODMAP diets on health is questioned due to its restrictive nature and potential negative effects on the gut microbiota [65,72]. There are also concerns related to nutritional deficiencies in an already predisposed population. In addition, the degree of symptoms does not always reflect the amount of inflammation in the intestines as measured by inflammation biomarkers [24].

This study has several strengths and is, to our knowledge, by far the most comprehensive systematic review conducted on dietary exposures and IBD-related outcomes to date. We have included different classifications and triangulated results classified by food groups, food patterns, and macronutrients, as well as biomarkers for disease. The triangulation enables different perspectives showing generally overlapping pictures. However, even if our search was extensive, there might have been sources missed due to inadequate indexing in the included databases, or titles and abstracts not indicating the articles to be relevant. An important criterium to evaluate exposures in association with IBD is to have sufficient follow-up time to observe differences in the outcomes of interest, such as incidence, remission, and progression. Generally, most studies had a sufficiently long time perspective to allow for this. The quality of the included systematic reviews and meta-analyses varied, with the majority evaluated to be of high quality, but also, many had lower quality. We also applied state-of-the art methodology in the conduction of this systematic review.

In conclusion, a high intake of fruits and vegetables are both strongly associated with reduced incidence of UC and CD. Also, Mediterranean, vegetarian, and semivegetarian diets are associated with reduced incidence and progression of IBD. In contrast, dietary patterns high in meat, ultraprocessed food, dietary fats, and refined sugars are associated with increased incidence of IBD, UC, and CD. Many of the associations are mirrored by studies on inflammatory markers, microbiota, and other mechanisms, increasing the level of certainty in causal effects. With the strong associations with both incidence and progression to disease of several dietary factors, it would be a lost opportunity in not providing dietary guidance as part of the management of IBD.

## Author contributions

The authors’ responsibilities were as follows—CC, AK, LTF: conceptualized the review and protocol; CC, AK, ISS: formulated the search string and adapted it for different databases and conducted the search in each database; CC, AK: screened titles and abstracts for inclusion and eligibility using Rayyan as a screening tool, and extracted data from all included studies; CC, AK, EKA, LTF: evaluated the quality of each study using AMSTAR-2; LTF: coded the results section of all quantitative studies and summarized them in figures; CC, AK, EKA, LTF: partook in discussions and decisions regarding data synthesis; CC, AK, EKA, JGH, ISS, LTF: contributed original writing to the draft; CC, AK, EKA, JGH, LTF: reviewed the draft for correct medical content; all authors: read and approved the final version of the manuscript.

## Conflicts of interest

The authors report no conflicts of interest.

### Funding

The authors were funded by their respective institutions. The study had no additional funding. The funders had no role in study design, data collection and analysis, decision to publish, or preparation of the manuscript.
