# Supplementary material for: Diet, Food, and Nutritional Exposures and Inflammatory Bowel Disease or Progression of Disease: an Umbrella Review
Source: Adv Nutr. 2024 Apr 8;15(5):100219. doi: 10.1016/j.advnut.2024.100219 (PMC11063602; doi:10.1016/j.advnut.2024.100219)
Supplement: Multimedia component 1 [file mmc1.docx]

**Diet, food, and nutritional exposures and inflammatory bowel disease or progression of disease: an umbrella review**

Christensen et al.

Online Supplementary material

**Supplementary file for umbrella review on diet and inflammatory bowel disease including the following items:**

**Supplementary Text S1:** Search strategy

**Supplementary Table S1:** Summary of quantitative data with first author, year, disease, exposure, measure, number of participants, cases, and quality grading.

**Supplementary Table S2:** Summary of qualitative findings from systematic review (for outcomes without meta-analysis) listed by first author and exposure.

**Supplementary Table S3:** Summary of biomarkers for disease listed by first author and exposure.

**Supplementary Table S4:** Summary of quality grading listed by first author and AMSTAR-2 item number with total grading.

**Supplementary Table S5:** Summary of specific dietary patterns.

**Supplementary Figure S1:** Summary of associations from the meta-analyses meat and meat products and incidence of inflammatory bowel disease (IBD), ulcerative colitis (UC) and Crohn’s' disease (CD).

**Supplementary Figure S2a-e:** Summary of associations from the meta-analyses on meat (a: total meat, b: red meat, c: processed meat, d: white meat, e: fish) and incidence of inflammatory bowel disease (IBD).

**Supplementary Figure S3a-e:** Summary of associations from the meta-analyses on meat (a: total meat, b: red and processed meat, c: red meat, d: processed meat, e: white meat, f: fish) and incidence of ulcerative colitis (UC).

**Supplementary Figure S4a-e:** Summary of associations from the meta-analyses on meat (a: total meat, b: red meat, c: processed meat, d: white meat, e: fish) and incidence of Crohn’s' disease (CD).

**Supplementary Figure S5:** Summary of associations from the meta-analyses between dietary exposures and relapse/progression of ulcerative colitis.

**Supplementary Figure S6:** Summary of associations from the meta-analyses between dietary exposures and relapse/progression of Crohn’s' disease.

**Supplementary Figure S7:** Summary of associations from the meta-analyses between dietary exposures and symptoms of inflammatory bowel disease.

**Supplementary Text S1: Search strategy**

**Ovid MEDLINE(R) and Epub Ahead of Print, In-Process, In-Data-Review & Other Non-Indexed Citations and Daily <1946 to June 13, 2023>**

**Date: 14.06.2023**

1 exp Inflammatory Bowel Diseases/ 96568

2 (inflammatory bowel disease* or IBD or ulcer* colitis or colitis ulcer* or morbus crohn or crohn's disease* or crohn disease*).ti,ab,kf. 124998

3 1 or 2 138144

4 "Diet, Food, and Nutrition"/ or Diet/ or Food/ or Diet Records/ 223295

5 (Diet* or food* or nutrition* or meal*).ti,ab,kf. 1424545

6 4 or 5 1464497

7 Meta-Analysis/ or Systematic Review/ 313213

8 (Systematic review* or meta-analy* or metaanaly* or meta analy*).ti,ab,kf. 421376

9 7 or 8 456732

10 3 and 6 and 9 319

11 exp animals/ not humans/ 5130332

**12 10 not 11 317**

<https://ovidsp.ovid.com/ovidweb.cgi?T=JS&NEWS=N&PAGE=main&SHAREDSEARCHID=18J2cqCONwVzP59hs9KYRAWA5nUqJUD1PcIoGHWLi8pLK5t1mbwWJWCzKt8eNsaG7>

**Embase <1974 to 2023 June 13>**

**Date: 14.06.2023**

1 exp Crohn disease/ or inflammatory bowel disease/ or ulcerative colitis/ or acute severe ulcerative colitis/ 196147

2 (inflammatory bowel disease* or IBD or ulcer* colitis or colitis ulcer* or morbus crohn or crohn's disease* or crohn disease*).ti,ab,kf. 207712

3 1 or 2 240281

4 diet/ or dietary intake/ or dietary pattern/ or food frequency questionnaire/ or food intake/ or nutrition/ 575623

5 (Diet* or food* or nutrition* or meal*).ti,ab,kf. 1763897

6 4 or 5 1873671

7 meta analysis/ or "systematic review"/ 569355

8 (Systematic review* or meta-analy* or metaanaly* or meta analy*).ti,ab,kf. 550650

9 7 or 8 702141

10 3 and 6 and 9 829

11 limit 10 to (conference abstract or conference paper or "conference review") 182

12 exp animal/ not exp human/ 5190913

**13 10 not (11 or 12) 639**

<https://ovidsp.ovid.com/ovidweb.cgi?T=JS&NEWS=N&PAGE=main&SHAREDSEARCHID=Oj5zfzY4Q55JZkmAPvuFhHDw91DmuKSL1F0zv2m1MMfuZGFKorPhNyg9hbVbojwd>

**Epistemonikos**

**Date: 14.06.2023**

(title:(inflammatory bowel disease* OR IBD OR ulcer* colitis OR colitis ulcer* OR morbus crohn OR crohn's disease* OR crohn disease*) OR abstract:(inflammatory bowel disease* OR IBD OR ulcer* colitis OR colitis ulcer* OR morbus crohn OR crohn's disease* OR crohn disease*)) AND (title:(Diet* OR food* OR nutrition* OR meal*) OR abstract:(Diet* OR food* OR nutrition* OR meal*))

With Filter Systematic Reviews: [[198](https://www.epistemonikos.org/advanced_search?q=(title:(inflammatory%20bowel%20disease*%20OR%20IBD%20OR%20ulcer*%20colitis%20OR%20colitis%20ulcer*%20OR%20morbus%20crohn%20OR%20crohn%27s%20disease*%20OR%20crohn%20disease*)%20OR%20abstract:(inflammatory%20bowel%20disease*%20OR%20IBD%20OR%20ulcer*%20colitis%20OR%20colitis%20ulcer*%20OR%20morbus%20crohn%20OR%20crohn%27s%20disease*%20OR%20crohn%20disease*))%20AND%20(title:(Diet*%20OR%20food*%20OR%20nutrition*%20OR%20meal*)%20OR%20abstract:(Diet*%20OR%20food*%20OR%20nutrition*%20OR%20meal*))&protocol=no&classification=systematic-review)](https://www.epistemonikos.org/advanced_search?q=(title:(inflammatory%20bowel%20disease*%20OR%20IBD%20OR%20ulcer*%20colitis%20OR%20colitis%20ulcer*%20OR%20morbus%20crohn%20OR%20crohn%27s%20disease*%20OR%20crohn%20disease*)%20OR%20abstract:(inflammatory%20bowel%20disease*%20OR%20IBD%20OR%20ulcer*%20colitis%20OR%20colitis%20ulcer*%20OR%20morbus%20crohn%20OR%20crohn%27s%20disease*%20OR%20crohn%20disease*))%20AND%20(title:(Diet*%20OR%20food*%20OR%20nutrition*%20OR%20meal*)%20OR%20abstract:(Diet*%20OR%20food*%20OR%20nutrition*%20OR%20meal*))&protocol=no&classification=systematic-review)

With Filter Broad Synthesis: [[15](https://www.epistemonikos.org/advanced_search?q=(title:(inflammatory%20bowel%20disease*%20OR%20IBD%20OR%20ulcer*%20colitis%20OR%20colitis%20ulcer*%20OR%20morbus%20crohn%20OR%20crohn%27s%20disease*%20OR%20crohn%20disease*)%20OR%20abstract:(inflammatory%20bowel%20disease*%20OR%20IBD%20OR%20ulcer*%20colitis%20OR%20colitis%20ulcer*%20OR%20morbus%20crohn%20OR%20crohn%27s%20disease*%20OR%20crohn%20disease*))%20AND%20(title:(Diet*%20OR%20food*%20OR%20nutrition*%20OR%20meal*)%20OR%20abstract:(Diet*%20OR%20food*%20OR%20nutrition*%20OR%20meal*))&protocol=no&classification=broad-synthesis)](https://www.epistemonikos.org/advanced_search?q=(title:(inflammatory%20bowel%20disease*%20OR%20IBD%20OR%20ulcer*%20colitis%20OR%20colitis%20ulcer*%20OR%20morbus%20crohn%20OR%20crohn%27s%20disease*%20OR%20crohn%20disease*)%20OR%20abstract:(inflammatory%20bowel%20disease*%20OR%20IBD%20OR%20ulcer*%20colitis%20OR%20colitis%20ulcer*%20OR%20morbus%20crohn%20OR%20crohn%27s%20disease*%20OR%20crohn%20disease*))%20AND%20(title:(Diet*%20OR%20food*%20OR%20nutrition*%20OR%20meal*)%20OR%20abstract:(Diet*%20OR%20food*%20OR%20nutrition*%20OR%20meal*))&protocol=no&classification=broad-synthesis)

With Filter Structured Summary: [[1](https://www.epistemonikos.org/advanced_search?q=(title:(inflammatory%20bowel%20disease*%20OR%20IBD%20OR%20ulcer*%20colitis%20OR%20colitis%20ulcer*%20OR%20morbus%20crohn%20OR%20crohn%27s%20disease*%20OR%20crohn%20disease*)%20OR%20abstract:(inflammatory%20bowel%20disease*%20OR%20IBD%20OR%20ulcer*%20colitis%20OR%20colitis%20ulcer*%20OR%20morbus%20crohn%20OR%20crohn%27s%20disease*%20OR%20crohn%20disease*))%20AND%20(title:(Diet*%20OR%20food*%20OR%20nutrition*%20OR%20meal*)%20OR%20abstract:(Diet*%20OR%20food*%20OR%20nutrition*%20OR%20meal*))&protocol=no&classification=structured-summary-of-systematic-review)](https://www.epistemonikos.org/advanced_search?q=(title:(inflammatory%20bowel%20disease*%20OR%20IBD%20OR%20ulcer*%20colitis%20OR%20colitis%20ulcer*%20OR%20morbus%20crohn%20OR%20crohn%27s%20disease*%20OR%20crohn%20disease*)%20OR%20abstract:(inflammatory%20bowel%20disease*%20OR%20IBD%20OR%20ulcer*%20colitis%20OR%20colitis%20ulcer*%20OR%20morbus%20crohn%20OR%20crohn%27s%20disease*%20OR%20crohn%20disease*))%20AND%20(title:(Diet*%20OR%20food*%20OR%20nutrition*%20OR%20meal*)%20OR%20abstract:(Diet*%20OR%20food*%20OR%20nutrition*%20OR%20meal*))&protocol=no&classification=structured-summary-of-systematic-review)

**Supplementary Table S1:** Summary of quantitative data with first author, year, disease, exposure, measure, number of participants, cases, and quality grading.

| **First author** | **Year** | **Disease** | **(specific)** | **Exposure** | **(scaling)** | **Measure** | **Risk measure** | **studies** | **n** | **cases** | **h2** | **Evidence** | **AMSTAR-2** |
| --- | --- | --- | --- | --- | --- | --- | --- | --- | --- | --- | --- | --- | --- |
| Talebi | 2023 | IBD | incidence | Red meat | HL | RR | 1.10 (0.97, 1.25) | 6 | 3666182 | 7383 | 56 | Low | high |
| Talebi | 2023 | IBD | incidence | Red meat | 100g | RR | 1.34 (0.96, 1.86) | 3 | 3666182 | 7383 | 33 | Low | high |
| Talebi | 2023 | CD | incidence | Red meat | HL | RR | 1.02 (0.82, 1.28) | 5 | 3666182 | 7383 | 67 |  | high |
| Talebi | 2023 | UC | incidence | Red meat | HL | RR | 1.16 (0.96, 1.40) | 5 | 3666182 | 7383 | 59 |  | high |
| Talebi | 2023 | IBD | incidence | Processed meat | HL | RR | 1.09 (0.94, 1.26) | 5 | 3583035 | 6824 | 61 | Very low | high |
| Talebi | 2023 | CD | incidence | Processed meat | HL | RR | 1.01 (0.78, 1.30) | 5 | 3583035 | 6824 | 66 |  | high |
| Talebi | 2023 | UC | incidence | Processed meat | HL | RR | 1.16 (0.99, 1.37) | 5 | 3583035 | 6824 | 32 |  | high |
| Talebi | 2023 | IBD | incidence | Poultry | HL | RR | 1.18 (0.88, 1.59) | 2 | 529677 | 1071 | 45 | Very low | high |
| Talebi | 2023 | IBD | incidence | Poultry | 100g | RR | 1.74 (0.49, 6.13) | 2 | 529677 | 1071 | 52 | Very low | high |
| Talebi | 2023 | CD | incidence | Poultry | HL | RR | 1.42 (0.87, 2.33) | 1 | 529677 | 1071 |  |  | high |
| Talebi | 2023 | UC | incidence | Poultry | HL | RR | 0.92 (0.67, 1.26) | 1 | 529677 | 1071 |  |  | high |
| Talebi | 2023 | IBD | incidence | Fish | HL | RR | 1.03 (0.92, 1.15) | 5 | 660429 | 4757 | 25 | Low | high |
| Talebi | 2023 | IBD | incidence | Fish | 15g | RR | 0.99 (0.88, 1.11) | 5 | 660429 | 4757 | 50 | Low | high |
| Talebi | 2023 | CD | incidence | Fish | HL | RR | 0.92 (0.73, 1.15) | 4 | 660429 | 4757 | 43 |  | high |
| Talebi | 2023 | UC | incidence | Fish | HL | RR | 1.07 (0.95, 1.20) | 4 | 660429 | 4757 | 0 |  | high |
| Talebi | 2023 | IBD | incidence | Meat and meat products | HL | RR | 1.24 (0.90, 1.70) | 4 | 535738 | 1214 | 67 | Low | high |
| Talebi | 2023 | IBD | incidence | Meat and meat products | 100g | RR | 1.38 (1.13, 1.68) | 3 | 535738 | 1214 | 0 | Low | high |
| Talebi | 2023 | CD | incidence | Meat and meat products | HL | RR | 1.28 (0.76, 2.16) | 1 | 535738 | 1214 |  |  | high |
| Talebi | 2023 | UC | incidence | Meat and meat products | HL | RR | 1.40 (0.99, 1.98) | 1 | 535738 | 1214 |  |  | high |
| Talebi | 2023 | IBD | incidence | Dairy | HL | RR | 0.81 (0.72, 0.90) | 7 | 1097040 | 7232 | 36 | Moderate | high |
| Talebi | 2023 | IBD | incidence | Dairy | 200g | RR | 0.97 (0.92, 1.01) | 6 | 1097040 | 7232 | 0 | Moderate | high |
| Talebi | 2023 | CD | incidence | Dairy | HL | RR | 0.69 (0.56, 0.86) | 5 | 1097040 | 7232 | 50 |  | high |
| Talebi | 2023 | UC | incidence | Dairy | HL | RR | 0.84 (0.75, 0.94) | 5 | 1097040 | 7232 | 0 |  | high |
| Talebi | 2023 | IBD | incidence | Eggs | HL | RR | 0.92 (0.81, 1.04) | 3 | 492497 | 4025 | 0 | Low | high |
| Talebi | 2023 | IBD | incidence | Eggs | 50g | RR | 0.99 (0.65, 1.52) | 3 | 492497 | 4025 | 0 | Low | high |
| Talebi | 2023 | CD | incidence | Eggs | HL | RR | 0.94 (0.78, 1.12) | 2 | 492497 | 4025 | 0 |  | high |
| Talebi | 2023 | UC | incidence | Eggs | HL | RR | 0.90 (0.76, 1.07) | 2 | 492497 | 4025 | 0 |  | high |
| Narula | 2023 | CD | incidence | Ultraprocessed food | HL | HR | 1.71 (1.36, 2.14) | 4 | 960638 | 889 | 0 | Low | high |
| Narula | 2023 | UC | incidence | Ultraprocessed food | HL | HR | 1.17 (0.86, 1.61) | 4 | 960638 | 1886 | 74 | Low | high |
| Narula | 2023 | CD | incidence | Minimally processed | HL | HR | 0.71 (0.53, 0.94) | 2 | 658702 | 548 | 11 | Low | high |
| Narula | 2023 | UC | incidence | Minimally processed | HL | HR | 0.84 (0.68, 1.02) | 2 | 658702 | 919 | 0 | Low | high |
| Limketkai | 2023 | CD | relapse/ progression | Highly restrictive, organic diet | vs. control | RR | 1.03 (0.59–1.80) | 6 | 14 | 11 |  | Very low | low |
| Limketkai | 2023 | CD | relapse/ progression | Low-refined carbohydrate diet | vs. control | RR | 7.35 (1.14–47.5) | 6 | 16 | 8 | 0 | Very low | low |
| Limketkai | 2023 | CD | relapse/ progression | Symptoms-guided diet | vs. control | RR | 20.0 (1.27–315 ) | 6 | 51 | 16 |  | Very low | low |
| Limketkai | 2023 | CD | relapse/ progression | Low microparticle | vs. control | RR | 3.13 (0.22–43.8) | 6 | 103 | 36 | 73 | Very low | low |
| Limketkai | 2023 | CD | relapse/ progression | Low calcium diet | vs. control | RR | 1.24 (0.67–2.29) | 6 | 83 | 28 |  | Very low | low |
| Limketkai | 2023 | CD | relapse/ progression | Specific Carbohydrate Diet | vs. MED | RR | 0.86 (0.63–1.19) | 6 | 191 | 83 |  | Low | low |
| Limketkai | 2023 | CD | relapse/ progression | Specific Carbohydrate Diet | whole food | RR | 1.00 (0.59–1.70) | 6 | 10 | 10 |  | Very low | low |
| Limketkai | 2023 | CD | relapse/ progression | Partial enteral nutrition | vs. control | RR | 0.75 (0.21–2.71) | 6 | 124 | 64 | 83 | Very low | low |
| Limketkai | 2023 | CD | relapse/ progression | Partial enteral nutrition | vs. control | RR | 1.20 (0.74–1.94) | 6 | 40 | 25 |  | Very low | low |
| Limketkai | 2023 | CD | relapse/ progression | Specific Carbohydrate Diet | vs. control | RR | 1.13 (0.42-3.04) | 6 | 36 | 12 |  |  | low |
| Limketkai | 2023 | CD | relapse/ progression | Partial enteral nutrition | vs. control | RR | 0.75 (0.31-1.83) | 6 | 97 | 17 | 0 |  | low |
| Limketkai | 2023 | CD | relapse/ progression | Highly restrictive, organic diet | vs. control | RR | 6.75 (0.98-46.6) | 6 | 13 | 4 |  |  | low |
| Limketkai | 2023 | CD | relapse/ progression | Partial enteral nutrition | vs. control | RR | 1.16 (0.54-2.46) | 6 | 28 | 14 |  |  | low |
| Limketkai | 2023 | CD | relapse/ progression | Red meat | vs. control | RR | 0.99 (0.80–1.23) | 6 | 202 | 126 |  | Low | low |
| Limketkai | 2023 | CD | relapse/ progression | Low-refined carbohydrate diet | vs. control | RR | 1.04 (0.87–1.25) | 6 | 567 | 369 | 35 | Low | low |
| Limketkai | 2023 | CD | relapse/ progression | Anti-inflammatory diet | vs. control | RR | 0.11 (0.01–1.76) | 6 | 54 | 10 |  | Very low | low |
| Limketkai | 2023 | CD | relapse/ progression | Partial enteral nutrition | vs. control | RR | 0.52 (0.38–0.72) | 6 | 283 | 106 | 8 | Very low | low |
| Limketkai | 2023 | CD | relapse/ progression | Symptoms-guided diet | vs. control | RR | 0.61 (0.22–1.69) | 6 | 96 | 55 | 78 | Very low | low |
| Limketkai | 2023 | CD | relapse/ progression | Partial enteral nutrition | vs. control | RR | 0.62 (0.32-1.21) | 6 | 78 | 70 | 66 |  | low |
| Limketkai | 2023 | UC | relapse/ progression | Symptoms-guided diet | vs. control | RR | 6.00 (0.37–97) | 6 | 18 | 4 |  | Very low | low |
| Limketkai | 2023 | UC | relapse/ progression | Dairy elimination | vs. control | RR | 1.22 (0.88–1.69) | 6 | 106 | 48 | 9 | Very low | low |
| Limketkai | 2023 | UC | relapse/ progression | Gluten-free diet | vs. control | RR | 1.42 (0.54–3.76) | 6 | 51 | 13 |  | Very low | low |
| Limketkai | 2023 | UC | relapse/ progression | Symptoms-guided diet | vs. control | RR | 2.18 (0.67-7.16) | 6 | 17 | 10 |  |  | low |
| Limketkai | 2023 | UC | relapse/ progression | Symptoms-guided diet | vs. control | RR | 0.55 (0.16-1.91) | 6 | 17 | 6 |  |  | low |
| Limketkai | 2023 | UC | relapse/ progression | Dairy elimination | vs. control | RR | 2.02 (1.08-3.76) | 6 | 71 | 43 |  |  | low |
| Limketkai | 2023 | UC | relapse/ progression | Gluten-free diet | vs. control | RR | 1.98 (1.03-3.79) | 6 | 46 | 25 |  |  | low |
| Limketkai | 2023 | UC | relapse/ progression | Dairy elimination | vs. control | RR | 2.13 (0.73-6.19) | 6 | 49 | 19 |  |  | low |
| Limketkai | 2023 | UC | relapse/ progression | Gluten-free diet | vs. control | RR | 1.81 (0.57-5.78) | 6 | 32 | 10 |  |  | low |
| Limketkai | 2023 | UC | relapse/ progression | Carrageenan-free diet | vs. control | RR | 0.11 (0.01–1.71) | 6 | 12 | 3 |  | Very low | low |
| Limketkai | 2023 | UC | relapse/ progression | Anti-inflammatory diet | vs. control | RR | 0.65 (0.24–1.73) | 6 | 53 | 13 |  | Very low | low |
| Limketkai | 2023 | UC | relapse/ progression | Dairy elimination | vs. control | RR | 0.86 (0.62–1.20) | 6 | 78 | 49 | 0 | Low | low |
| Limketkai | 2023 | UC | relapse/ progression | Anti-inflammatory diet | vs. control | RR | 0.49 (0.26-0.93) | 6 | 53 | 25 |  |  | low |
| Zhao | 2022 | CD | incidence | Fiber | HL | OR | 0.68 (0.57-0.82) | 9 | 212851 | 1039 | 50 |  | low |
| Zhao | 2022 | CD | incidence | Breastfeeding | HL | OR | 0.81 (0.70-0.94) | 26 | 415566 | 7601 | 78 |  | low |
| Zhao | 2022 | CD | incidence | Fruits and vegetables | HL | OR | 0.66 (0.58-0.75) | 15 | 228639 | 1860 | 48 |  | low |
| Zhao | 2022 | CD | incidence | Vegetables | HL | OR | 0.74 (0.63-0.87) | 18 | 230452 | 1976 | 54 |  | low |
| Zhao | 2022 | CD | incidence | Eggs | HL | OR | 1.15 (0.92-1.44) | 9 | 15449 | 855 | 32 |  | low |
| Zhao | 2022 | CD | incidence | Fish | HL | OR | 0.67 (0.52-0.87) | 10 | 54953 | 1009 | 72 |  | low |
| Zhao | 2022 | CD | incidence | Meat and meat products | HL | OR | 1.59 (1.24-2.04) | 8 | 16454 | 1545 | 82 |  | low |
| Zhao | 2022 | CD | incidence | Alcohol | HL | OR | 0.91 (0.72-1.13) | 11 | 14337942 | 2748 | 75 |  | low |
| Zhao | 2022 | CD | incidence | Coffee | HL | OR | 0.71 (0.50-1.02) | 6 | 3268 | 934 | 71 |  | low |
| Zhao | 2022 | CD | incidence | Sugar-sweetened beverages | HL | OR | 1.50 (1.21-1.86) | 10 | 88837 | 1954 | 56 |  | low |
| Zhao | 2022 | CD | incidence | Dairy | HL | OR | 0.80 (0.67-0.97) | 10 | 16505 | 1254 | 63 |  | low |
| Zhao | 2022 | UC | incidence | Breastfeeding | HL | OR | 0.84 (0.75-0.94) | 24 | 415118 | 7245 | 63 |  | low |
| Zhao | 2022 | UC | incidence | Fiber | HL | OR | 1.01 (0.90-1.12) | 10 | 213947 | 1285 | 0 |  | low |
| Zhao | 2022 | UC | incidence | Fruits and vegetables | HL | OR | 0.76 (0.64-0.90) | 13 | 230679 | 2465 | 70 |  | low |
| Zhao | 2022 | UC | incidence | Vegetables | HL | OR | 0.82 (0.68-0.98) | 18 | 232966 | 3878 | 78 |  | low |
| Zhao | 2022 | UC | incidence | Eggs | HL | OR | 1.25 (1.03-1.52) | 11 | 15808 | 1093 | 85 |  | low |
| Zhao | 2022 | UC | incidence | Fish | HL | OR | 1.05 (0.88-1.26) | 9 | 59157 | 669 | 15 |  | low |
| Zhao | 2022 | UC | incidence | Meat and meat products | HL | OR | 1.14 (0.99-1.30) | 10 | 15670 | 1351 | 50 |  | low |
| Zhao | 2022 | UC | incidence | Alcohol | HL | OR | 0.86 (0.73-1.01) | 16 | 287148 | 3929 | 70 |  | low |
| Zhao | 2022 | UC | incidence | Coffee | HL | OR | 0.58 (0.43-0.79) | 8 | 3994 | 1378 | 73 |  | low |
| Zhao | 2022 | UC | incidence | Sugar-sweetened beverages | HL | OR | 1.16 (1.00-1.34) | 8 | 88504 | 1687 | 32 |  | low |
| Zhao | 2022 | UC | incidence | Dairy | HL | OR | 0.91 (0.78-1.06) | 10 | 18434 | 2292 | 22 |  | low |
| Peng | 2022 | IBD | symptoms | Low-FODMAP | vs. control | RR | 0.47 (0.33, 0.66) | 9 | 681 | 342 | 84 |  | high |
| Peng | 2022 | IBD | symptoms | Low-FODMAP | vs. control | RR | 0.44 (0.34, 0.55) | 7 | 323 | 162 | 0 |  | high |
| Peng | 2022 | IBD | symptoms | Low-FODMAP | vs. control | RR | 0.43 (0.33, 0.56) | 6 | 257 | 127 | 22 |  | high |
| Peng | 2022 | IBD | symptoms | Low-FODMAP | vs. control | RR | 0.50 (0.37, 0.68) | 5 | 548 | 272 | 55 |  | high |
| Peng | 2022 | IBD | symptoms | Low-FODMAP | vs. control | RR | 0.54 (0.22, 1.32) | 4 | 470 | 235 | 87 |  | high |
| Peng | 2022 | IBD | symptoms | Low-FODMAP | vs. control | RR | 0.37 (0.24, 0.57) | 4 | 548 | 272 | 70 |  | high |
| Peng | 2022 | IBD | symptoms | Low-FODMAP | vs. control | RR | 0.38 (0.28, 0.51) | 3 | 390 | 195 | 14 |  | high |
| Peng | 2022 | IBD | symptoms | Low-FODMAP | vs. control | RR | 0.48 (0.26, 0.89) | 3 | 326 | 163 | 69 |  | high |
| Peng | 2022 | IBD | symptoms | Low-FODMAP | vs. control | RR | 0.71 (0.61, 0.82) | 4 | 470 | 235 | 10 |  | high |
| Peng | 2022 | IBD | symptoms | Low-FODMAP | vs. control | MD | -0.4 (-0.5, -0.3) | 3 | 298 | 150 | 0 |  | high |
| Peng | 2022 | IBD | symptoms | Low-FODMAP | vs. control | MD | -93 (-144, -42 ) | 2 | 116 | 58 | 69 |  | high |
| Peng | 2022 | IBD | symptoms | Low-FODMAP | vs. control | MD | 11.2 (6.61, 15.9) | 2 | 166 | 83 | 0 |  | high |
| Peng | 2022 | IBD | QoL | Low-FODMAP | vs. control | MD | -3.7 (-22 , 14.7) | 2 | 170 | 85 | 0 |  | high |
| Peng | 2022 | IBD | symptoms | Low-FODMAP | vs. control | RR | 5.99 (0.17, 216 ) | 2 | 262 | 131 | 92 |  | high |
| Peng | 2022 | IBD | symptoms | Low-FODMAP | vs. control | MD | -0.2 (-0.5, 0.15) | 2 | 228 | 115 | 0 |  | high |
| Peng | 2022 | IBD | symptoms | Low-FODMAP | vs. control | MD | -0.3 (-1.1, 0.45) | 2 | 59 | 29 | 57 |  | high |
| Peng | 2022 | IBD | symptoms | Low-FODMAP | vs. control | MD | -1.1 (-1.8, -0.4) | 3 | 97 | 48 | 43 |  | high |
| Sinopoulou | 2021 | IBD | symptoms | Low-FODMAP | vs. control | MD | -4.0 (-21 , 12.6) | 1 | 26 | 13 |  | Very low | high |
| Sinopoulou | 2021 | IBD | symptoms | Low-FODMAP | vs. control | MD | -9.0 (-20 , 2.07) | 1 | 26 | 13 |  | Very low | high |
| Sinopoulou | 2021 | IBD | symptoms | Kefir | vs. control | MD | -0.2 (-0.9, 0.57) | 1 | 25 | 15 |  | Very low | high |
| Sinopoulou | 2021 | IBD | symptoms | Low-FODMAP | vs. control | MD | -2.0 (-16 , 11.9) | 1 | 52 | 27 |  | Very low | high |
| Sinopoulou | 2021 | IBD | symptoms | Low-FODMAP | vs. control | MD | 0.40 (-0.44, 1.24) | 1 | 52 | 27 |  | Very low | high |
| Sinopoulou | 2021 | IBD | symptoms | Low-FODMAP | vs. control | MD | -12 (-115, 90.6) | 1 | 26 | 14 |  | Very low | high |
| Sinopoulou | 2021 | IBD | symptoms | Low-FODMAP | vs. control | MD | -8.5 (-16 , -1.2) | 2 | 82 | 37 |  | Very low | high |
| Sinopoulou | 2021 | IBD | symptoms | Low-FODMAP | vs. control | MD | 0.20 (-8.67, 9.07) | 1 | 52 | 27 |  | Very low | high |
| Sinopoulou | 2021 | IBD | symptoms | Low-FODMAP | vs. control | MD | -8.0 (-66 , 50.3) | 1 | 26 | 14 |  | Very low | high |
| Sinopoulou | 2021 | IBD | symptoms | Kefir | vs. control | MD | 0.62 (0.17, 1.07) | 1 | 48 | 28 |  | Very low | high |
| Sinopoulou | 2021 | IBD | symptoms | Kefir | vs. control | MD | -1.1 (-1.7, -0.5) | 1 | 20 | 10 |  | Very low | high |
| Shahinfar | 2021 | IBD | relapse/ progression | Curcumin | vs. control | SMD (%) | 0.86 (0.16, 1.56) | 7 |  |  | 90 |  | high |
| Shahinfar | 2021 | IBD | relapse/ progression | Curcumin | vs. control | SMD (%) | 0.51 (0.16, 0.85) | 6 |  |  | 52 |  | high |
| Shahinfar | 2021 | IBD | relapse/ progression | Curcumin | vs. control | SMD (%) | 0.74 (0.22, 1.26) | 3 |  |  | 56 |  | high |
| Shahinfar | 2021 | IBD | QoL | Curcumin | vs. control | SMD (%) | 1.23 (0.72, 1.74) | 1 |  |  |  |  | high |
| Milajerdi | 2021 | UC | incidence | Fruits and vegetables | HL | RR | 0.46 (0.33, 0.65) | 2 |  |  | 90 |  | critically |
| Milajerdi | 2021 | CD | incidence | Fruits and vegetables | HL | RR | 0.41 (0.32, 0.54) | 2 |  |  | 43 |  | critically |
| Milajerdi | 2021 | UC | incidence | Fiber | HL | RR | 1.34 (0.99, 1.80) | 5 |  |  | 0 |  | critically |
| Milajerdi | 2021 | CD | incidence | Fiber | HL | RR | 0.59 (0.43, 0.81) | 4 |  |  | 0 |  | critically |
| Khademi | 2021 | UC | incidence | Sugar | HL | RR | 1.59 (1.15–2.20) | 4 |  |  | 0 |  | moderate |
| Khademi | 2021 | CD | incidence | Sugar | HL | RR | 1.90 (1.06–3.41) | 4 |  |  | 58 |  | moderate |
| Khademi | 2021 | IBD | incidence | Sugar | HL | RR | 1.71 (1.24,2.38) | 4 |  |  | 42 |  | moderate |
| Khademi | 2021 | UC | incidence | Sugar-sweetened beverages | HL | RR | 1.02 (0.92–1.12) | 3 |  |  | 14 |  | moderate |
| Khademi | 2021 | CD | incidence | Sugar-sweetened beverages | HL | RR | 1.22 (0.91–1.64) | 4 |  |  | 75 |  | moderate |
| Khademi | 2021 | IBD | incidence | Sugar-sweetened beverages | HL | RR | 1.02 (0.94, 1.11) | 3 |  |  | 19 |  | moderate |
| Comeche | 2021 | CD | relapse/ progression | Prescription of a predefined diet | post vs pre | MD | -90 (-104,-76 ) | 7 | 165 | 165 | 96 |  | high |
| Comeche | 2021 | CD | relapse/ progression | Prescription of a predefined diet | post vs pre | MD | -108 (-182.1, -33 ) | 7 | 165 | 165 | 96 |  | high |
| Mozaffari | 2020 | IBD | incidence | Fish | HL | RR | 0.93 (0.85, 1.03) | 6 | 41601 | 823 | 76 |  | high |
| Mozaffari | 2020 | IBD | incidence | Fish | HL | RR | 0.68 (0.46, 1.00) | 6 | 41601 | 823 | 76 |  | high |
| Mozaffari | 2020 | CD | incidence | Fish | HL | RR | 0.94 (0.85, 1.04) | 5 | 41601 | 563 | 81 |  | high |
| Mozaffari | 2020 | CD | incidence | Fish | HL | RR | 0.54 (0.31, 0.96) | 5 | 41601 | 563 | 81 |  | high |
| Mozaffari | 2020 | UC | incidence | Fish | HL | RR | 0.82 (0.56, 1.22) | 4 | 41601 | 260 | 31 |  | high |
| Mozaffari | 2020 | IBD | incidence | Omega-3 | HL | RR | 1.05 (0.84, 1.32) | 6 | 172428 | 1102 | 57 |  | high |
| Mozaffari | 2020 | IBD | incidence | Omega-3 | HL | RR | 1.17 (0.80, 1.72) | 6 | 172428 | 1102 | 57 |  | high |
| Mozaffari | 2020 | CD | incidence | Omega-3 | HL | RR | 1.95 (1.06, 3.61) | 2 | 172428 | 468 | 80 |  | high |
| Mozaffari | 2020 | CD | incidence | Omega-3 | HL | RR | 1.62 (0.38, 6.87) | 2 | 172428 | 468 | 80 |  | high |
| Mozaffari | 2020 | UC | incidence | Omega-3 | HL | RR | 0.96 (0.75, 1.22) | 4 | 172428 | 634 | 0 |  | high |
| Mozaffari | 2020 | IBD | incidence | Omega-3 | HL | RR | 0.78 (0.63, 0.97) | 5 | 171983 | 868 | 0 |  | high |
| Mozaffari | 2020 | CD | incidence | Omega-3 | HL | RR | 0.85 (0.59, 1.23) | 2 | 171983 | 342 | 0 |  | high |
| Mozaffari | 2020 | UC | incidence | Omega-3 | HL | RR | 0.75 (0.57, 0.98) | 3 | 171983 | 526 | 0 |  | high |
| Mozaffari | 2020 | IBD | incidence | Dietary ALA intake | HL | RR | 1.17 (0.63, 2.17) | 3 | 171983 | 868 | 0 |  | high |
| Li | 2020 | IBD | incidence | Western diet | HL | RR | 1.92 (1.37, 2.68) | 9 | 54581 | 1492 | 70 |  | high |
| Li | 2020 | CD | incidence | Western diet | HL | RR | 1.72 (1.01, 2.93) | 7 | 53796 | 707 | 75 |  | high |
| Li | 2020 | UC | incidence | Western diet | HL | RR | 2.15 (1.38, 3.34) | 8 | 53873 | 784 | 66 |  | high |
| Li | 2020 | CD | incidence | Fast food | HL | RR | 1.85 (0.66, 5.19) | 3 | 53873 | 784 | 83 |  | high |
| Li | 2020 | UC | incidence | Fast food | HL | RR | 2.90 (1.04, 8.09) | 3 | 53873 | 784 | 83 |  | high |
| Li | 2020 | CD | incidence | Western diet | HL | RR | 1.69 (0.83, 3.47) | 4 | 53873 | 784 | 72 |  | high |
| Li | 2020 | UC | incidence | Western diet | HL | RR | 1.85 (1.09, 3.14) | 5 | 53873 | 784 | 66 |  | high |
| Khorshidi | 2020 | CD | incidence | Healthy/prudent dietary patterns | HL | OR | 0.39 (0.16, 0.62) | 5 | 223572 | 753 | 68 |  | low |
| Khorshidi | 2020 | UC | incidence | Western diet | HL | OR | 0.97 (0.67, 1.26) | 3 | 40693 | 400 | 0 |  | low |
| Khorshidi | 2020 | CD | incidence | Western diet | HL | OR | 0.78 (0.51, 1.04) | 6 | 223572 | 753 | 31 |  | low |
| Limketkai | 2019 | CD | relapse/ progression | High-fiber, low-refined carbohydrate diet | vs. control | RR | 7.20 (0.53, 97.83) | 6 | 7 |  |  | Very low | high |
| Limketkai | 2019 | CD | relapse/ progression | Low microparticle diet | vs. control | RR | 3.13 (0.22, 43.84) | 6 | 103 |  | 73 | Very low | high |
| Limketkai | 2019 | CD | relapse/ progression | Low calcium diet | vs. control | RR | 1.24 (0.67, 2.29) | 6 | 83 |  |  | Very low | high |
| Limketkai | 2019 | CD | relapse/ progression | Symptoms-guided diet | vs. control | RR | 20.0 (1.27, 315.) | 6 | 51 |  |  | Very low | high |
| Limketkai | 2019 | CD | relapse/ progression | Restricted organic diet | vs. control | RR | 1.00 (0.39, 2.53) | 6 | 18 |  |  | Very low | high |
| Limketkai | 2019 | IBD | QoL | IBDQ-symptoms-guided diet | vs. control | MD | 23.8 (7.12, 40.4) | 6 | 51 |  |  | Low | high |
| Limketkai | 2019 | IBD | QoL | IBDQ-highly restricted, organic diet | vs. control | MD | 4.00 (-17 , 25.9) | 6 | 14 |  |  | Very low | high |
| Limketkai | 2019 | CD | relapse/ progression | Low-refined carbohydrate diet | vs. control | RR | 1.04 (0.87, 1.25) | 6 | 567 |  | 35 | Low | high |
| Limketkai | 2019 | CD | relapse/ progression | Symptoms-guided diet | vs. control | RR | 0.53 (0.28, 1.01) | 6 | 98 |  | 54 | Low | high |
| Limketkai | 2019 | CD | relapse/ progression | Low red, processed meat diet | vs. control | RR | 1.03 (0.85, 1.26) | 6 | 214 |  |  | Low | high |
| Limketkai | 2019 | CD | relapse/ progression | Low sugars, grains, sat. fats, red & proc. meats | vs. control | RR | 0.11 (0.01, 1.76) | 6 | 54 |  |  | Very low | high |
| Limketkai | 2019 | UC | relapse/ progression | Symptoms-guided diet | vs. control | RR | 8.25 (0.50, 136) | 6 | 21 |  |  | Very low | high |
| Limketkai | 2019 | UC | relapse/ progression | Anti-inflammatory diet | vs. control | RR | 1.25 (0.42, 3.70) | 6 | 28 |  |  | Very low | high |
| Limketkai | 2019 | UC | relapse/ progression | Carrageenan-free diet | vs. control | RR | 0.50 (0.15, 1.64) | 6 | 15 |  |  | Very low | high |
| Limketkai | 2019 | UC | relapse/ progression | Milk free diet | vs. control | RR | 0.83 (0.60, 1.15) | 6 | 77 |  | 0 | Low | high |
| Limketkai | 2019 | IBD | QoL | Carrageenan-free diet | vs. control | MD | -1.7 (-8.2, 4.83) | 6 | 12 |  |  | Very low | high |
| Zhan | 2018 | IBD | symptoms | Low-FODMAP | vs. control | OR | 0.24 (0.11, 0.52) | 6 | 549 | 274 | 59 |  | low |
| Zhan | 2018 | IBD | symptoms | Low-FODMAP | vs. control | OR | 0.24 (0.16, 0.35) | 4 | 507 | 253 | 58 |  | low |
| Zhan | 2018 | IBD | symptoms | Low-FODMAP | vs. control | OR | 0.17 (0.12, 0.26) | 4 | 507 | 253 | 89 |  | low |
| Zhan | 2018 | IBD | symptoms | Low-FODMAP | vs. control | OR | 0.51 (0.31, 0.85) | 3 | 418 | 209 | 48 |  | low |
| Zhan | 2018 | IBD | symptoms | Low-FODMAP | vs. control | OR | 0.40 (0.24, 0.66) | 3 | 346 | 209 | 0 |  | low |
| Zhan | 2018 | IBD | symptoms | Low-FODMAP | vs. control | OR | 0.77 (0.44, 1.37) | 3 | 418 | 209 | 0 |  | low |
| Nie | 2017 | UC | incidence | Alcohol | HL | RR | 0.95 (0.65–1.39) | 9 | 339028 | 3689 | 67 |  | high |
| Nie | 2017 | UC | incidence | Coffee | HL | RR | 0.58 (0.33–1.05) | 6 | 339028 | 3689 | 88 |  | high |
| Nie | 2017 | UC | incidence | Sugar | HL | RR | 1.69 (1.24–2.30) | 5 | 339028 | 3689 | 13 |  | high |
| Nie | 2017 | UC | incidence | Tea | HL | RR | 0.69 (0.58–0.83) | 3 | 339028 | 3689 | 0 |  | high |
| Wu | 2016 | IBD | incidence | Fat | HL | RR | 1.52 (1.16-1.99) | 13 | 1084 |  | 0 |  | low |
| Wu | 2016 | UC | incidence | Fat | HL | RR | 1.46 (1.02, 2.09) | 6 | 480 |  | 0 |  | low |
| Wu | 2016 | CD | incidence | Fat | HL | RR | 1.57 (0.90, 2.74) | 5 | 440 |  | 17 |  | low |
| Liu | 2015 | CD | incidence | Fiber | HL | RR | 0.44 (0.29, 0.69) | 7 |  |  | 56 |  | low |
| Liu | 2015 | UC | incidence | Fiber | HL | RR | 0.80 (0.64, 1.00) | 8 |  |  | 48 |  | low |
| Li | 2015 | UC | incidence | Vegetables | HL | OR | 0.71 (0.58, 0.88) | 9 |  |  | 42 |  | low |
| Li | 2015 | CD | incidence | Vegetables | HL | OR | 0.66 (0.40, 1.09) | 8 |  |  | 68 |  | low |
| Li | 2015 | UC | incidence | Fruits and vegetables | HL | OR | 0.69 (0.49, 0.96) | 8 |  |  | 51 |  | low |
| Li | 2015 | CD | incidence | Fruits and vegetables | HL | OR | 0.57 (0.44, 0.74) | 10 |  |  |  |  | low |
| Ge | 2015 | IBD | incidence | Meat and meat products | HL | RR | 1.50 (1.15, 1.95) | 9 |  |  | 60 |  | low |
| Ge | 2015 | UC | incidence | Meat and meat products | HL | RR | 1.47 (1.01, 2.15) | 7 |  |  | 67 |  | low |
| Ge | 2015 | CD | incidence | Meat and meat products | HL | RR | 1.50 (0.98, 2.28) | 4 |  |  | 48 |  | low |
| Ge | 2015 | IBD | incidence | Red meat | HL | RR | 2.37 (1.40, 3.99) | 3 |  |  |  |  | low |
| Ge | 2015 | IBD | incidence | White meat | HL | RR | 1.20 (0.73, 1.97) | 2 |  |  |  |  | low |
| Ge | 2015 | IBD | incidence | Processed meat | HL | RR | 1.60 (0.53, 4.78) | 2 |  |  |  |  | low |

**Supplementary Table S2:** Summary of qualitative findings from systematic review (for outcomes without meta-analysis) listed by first author and exposure.

| **First author** | **Year** | **Studies (n)** | **Disease** | **Specific** | **Exposure** | **Findings** |
| --- | --- | --- | --- | --- | --- | --- |
| Limketkai | 2023 | 2 | IBD | Quality of life | Sequential elimination diet | Significantly increased quality of life scores |
| Limketkai | 2023 | 2 | CD | Quality of life | High-fiber, low-refined carbohydrate diet | Significantly increased quality of life scores |
| Limketkai | 2023 | 1 | CD | Quality of life | Specific Carbohydrate Diet (SCD) vs Mediterranean diet | Both led to improvement in quality of life scores |
| Limketkai | 2023 | 4 | UC | Quality of life | Low-fat, high-fiber diet vs. improved standard American diet | Both improved quality of life scores, but the change was greater for the low-fat, high-fiber diet arm |
| Limketkai | 2023 | 1 | UC | Quality of life | Carrageenan-free diet | No significant change in quality of life |
| Limketkai | 2023 | 1 | UC | Quality of life | Microparticle-free diet | No significant change in quality of life |
| Limketkai | 2023 | 1 | CD | Quality of life | Highly restrictive organic diet | No significant change in quality of life |
| Limketkai | 2023 | 2 | CD | Quality of life | IgG-driven exclusion diet | No significant change in quality of life |
| Limketkai | 2023 | 1 | CD | Quality of life | Anti-inflammatory diet | No significant change in quality of life |
| Cooper | 2023 | 1 | CD | Progression | Liquid diet | No significant symptom improvement. |
| Peters | 2022 | 2 | IBD | Progression | Germinated Barley Foodstuff (GBF) | Reduced clinical and biochemical disease markers, but sparse evidence |
| Peters | 2022 | 1 | UC | Progression | Inulin | Decreased disease activity scores |
| Peters | 2022 | 2 | IBD | Progression | Wheat bran | No improvement in objective clinical end points |
| Peters | 2022 | 3 | IBD | Progression | Psyllium | Conflicting evidence, some found less progression and some did not |
| Peters | 2022 | 1 | IBD | Progression | Fructo-oligosaccharides (FOS) | Lower IBD questionnaire scores (lower well-being) |
| Marsh | 2022 | 10 | UC | Progression | Elimination diet | Low quality studies recommend, but additional high quality studies needed |
| Jaber | 2022 | 5 | CD | Progression | Mediterranean Diet (MD) | Associated with improvement of symptoms and biomarkers |
| Irrazabal | 2021 | 4 | UC | Progression | Curcumin | Improve clinical activity |
| Wagenaar | 2021 | 1 | IBD | Progression | Low-fiber | increased disease activity |
| Wagenaar | 2021 | 3 | IBD | Progression | Supplemental fiber | Could decrease disease activity |
| Wagenaar | 2021 | 3 | IBD | Progression | High-fiber | Decreased disease-specific outcomes and CRP |
| Valido | 2021 | 3 | IBD | GI-symptoms | Oat | Conflicting evidence, significantly higher diarrhea in some weeks with oat in one study, but no increase in GI-symptoms in another. One found that low consumption predisposed the onset of CD. |
| Tian | 2021 | 1 | UC | Incidence | DII-score | High DII-score was associated with increased risk of UC |
| Tian | 2021 | 1 | CD | Incidence | EDIP-score | High EDIP-score was associated with increased risk of CD, but not UC |
| Tian | 2021 | 3 | IBD | Incidence | Mediterranean Diet (MD) | Conflicting evidence in correlation between MD and UC/CD. One found no correlation, another found decreased risk of CD, but unchanged risk of UC, and another found strong correlation between low MD and risk of UC. |
| Tian | 2021 | 3 | IBD | Progression | Mediterranean Diet (MD) | Lowered fecal calprotectin in UC, Harvey Bradshaw index and CRP. Positive correlation with the IBD-questionnaire |
| Tian | 2021 | 3 | IBD | GI-symptoms | DII/EDIP | Conflicting evidence. One study found that increased EDII was associated with rise in IBD-symptoms, another found that DII/EDIP did not. |
| Tian | 2021 | 1 | IBD | Calprotectin | DII/EDIP | Increased EDII was associated with greater odds of fecal calprotectin, but no relation was found between changes in DII and changes in fecal calprotectin or the IBD Symptom Index. |
| Comeche | 2021 |  | IBD | Progression | Low-FODMAP diet | Decreased GI-symptoms, not always improvement of biomarkers |
| Comeche | 2021 |  | IBD | Progression | The Specific Carbohydrate Diet (SCD) | Improved symptomatology and increase in clinical remission |
| Comeche | 2021 |  | IBD | Progression | The Immunoglobulin Exclusion Diet (IGED) | Contradictory results |
| Comeche | 2021 |  | IBD | Progression | Vegetarian | Associated with an decrease in CRP and other inflammatory markers |
| Comeche | 2021 |  | IBD | Progression | Mediterranean Diet (MD) | Positive results obtained regarding QoL, FC and CRP |
| Comeche | 2021 |  | IBD | Progression | Gluten-free diet | Improved symptoms |
| Mozaffari | 2020 | 3 | CD | Incidence | Fish | Inverse association between fish consumption and the risk of CD |
| Mozaffari | 2020 | 1 | UC | Incidence | Fish | Negative association between fish consumption and the risk of UC |
| Mozaffari | 2020 | 3 | IBD | Incidence | Fish | No association between fish consumption and the risk of IBD |
| Mozaffari | 2020 | 1 | CD | Incidence | Seafood | Positive association between seafood consumption and the risk of CD |
| Mozaffari | 2020 | 1 | IBD | Incidence | Seafood | Positive association between seafood consumption and the risk of IBD |
| Mozaffari | 2020 | 5 | IBD | Incidence | n-3 PUFA | Conflicting evidence |
| Montroy | 2020 | 2 | Active UC | Progression | Germinated Barley Foodstuff (GBF) | Significant decrease in clinical activity index |
| Montroy | 2020 | 3 | UC in remission | Progression | Resistant starches (RS) | Patients remained in remission |
| Coelho | 2020 | 6 | UC | Progression | Curcumin | May be effective in maintaining remission when combined with standard treatments, but more research is needed |
| Coelho | 2020 | 6 | CD | Progression | Curcumin | More research is needed |
| Rocha | 2019 | 2 | IBD | Progression | Malnutrition | Associated with hospitalization |
| Rocha | 2019 | 3 | IBD | Progression | Obesity | Disagreement between the studies in which obesity was associated with hospitalization in patients with IBD. |
| Lamb | 2019 | 5 | IBD | Progression | Dietary advice | Patients are advised to eat a varied diet meeting energy and nutritional needs, the diet should be based on healthy eating guidelines. It should include a wide variety of fruits and vegetables, grains, cereals, nuts and seeds, protein-rich foods with a reduction of fat, particularly animal fat, high sugar and processed meats. |
| Lamb | 2019 |  | IBD | Progression | fiber | May consider limiting fiber in diet, and supplementing with enteral or parenteral nutrition to achieve energy and nutritional requirements |
| Lamb | 2019 | 3 | IBD | Symptoms | Low-FODMAP diet | Symptom improvement for patient with functional GI-symptoms |
| Lamb | 2019 |  | IBD | Progression | Electrolytes | To prevent dehydration, sprinkle extra salt onto meals, increase potassium intake (bananas, potatoes, spinach, fish etc.) |
| Lamb | 2019 |  | IBD | Progression | Dietary advice | Small frequent, nutrient dense meals/snacks and oral nutritional supplements where necessary, recommend chewing food thoroughly and advise caution with fruit and vegetable skins, sweetcorn and celery and avoid nuts (smooth nut butters are fine). Monitor vit B12. |
| Lamb | 2019 |  | IBD | Progression | fiber | Caution is advised, high fiber intake may increase loose stools, flatulence and bloating |
| Güngör | 2019 | 13 | IBD | Incidence | Human milk (never vs. ever) | Higher risk of CD and UC in 3/4, and lower risk of CD in 1/4 of the studies |
| Güngör | 2019 | 9 | IBD | Incidence | Human milk (shorter vs. longer duration) | Higher risk of CD and UC |
| Franqui | 2019 | 2 | CD | Progression | The Specific Carbohydrate Diet (SCD) | Effective in inducing remission in pediatric patients, effect in adults too, but evidence is limited |
| Franqui | 2019 | 1 | IBD | Symptoms | Low-FODMAP diet | Improvement of GI-symptoms |
| Franqui | 2019 | 1 | CD | Progression | Gluten-free diet | insufficient evidence |
| Franqui | 2019 | 1 | CD | Progression | Low-residue diet | One study showed those receiving the highest proportions of low-residue meals had a significantly lower risk of CD-relapse, but there is lack of substantial evidence on the long-term use of this diet in CD patients. |
| Franqui | 2019 | 2 | CD | Progression | IgG4-based exclusion | May reduce symptoms and inflammation |
| McCormick | 2017 | 8 | CD | Progression | The Specific Carbohydrate Diet (SCD) | Associated with significant clinical improvements, including achievement and maintenance of remission from active disease |
| Szilagyi | 2016 | 14 | IBD | Progression | Dairy products | Conflicting evidence, may decrease the risk of IBD and disease activity. |
| Penagini | 2016 | 53 | IBD | Incidence | Dietary patterns with meat, fatty foods, desserts and high sugar in children | Associated with increased risk of IBD. Exclusion diets like the specific carbohydrate diet and crohns disease exclusion diet are showing promising data, however, it should be considered risk of nutritional deficits. |
| Charlebois | 2015 | 1 | CD | GI-symptoms | Low residue diet | No difference between groups |
| Charlebois | 2015 | 1 | CD | Progression | Carbohydrates (refined form with unrestricted sugar intake) vs. unrefined only | No difference between groups |
| Charlebois | 2015 | 1 | CD | Progression | Exclusion diet (intolerant foods) vs. unrefined carbohydrate fiber-rich diet | Longer time to relapse in control group, nonsignificant improvement in patient’s erythrocyte sedimentation rate (ESR) and orosomucoid concentrations. |
| Charlebois | 2015 | 1 | CD | Progression | Elemental diet vs. corticosteroids | Significant improvements in diet group in ESR, serum-albumin, and CRP. |
| Charlebois | 2015 | 1 | CD | GI-symptoms | Exclusion diet | Significant improvement in symptoms in diet group |
| Charlebois | 2015 | 1 | CD | Progression | IgG-based exclusion | Significant improvement in modified CD activity index (mCDAI) scores. Non-significant reduction in ESR, no significant change in CRP or albumin levels. |
| Charlebois | 2015 | 1 | CD | GI-symptoms | IgG-based exclusion | Significantly lower stool frequency in elimination diet group |
| Charlebois | 2015 | 1 | CD | Progression | A diet low in ingested matter from environmental factors vs. low-fat and high-carbohydrate control diet | Significant improvement in MRI/endoscopy evaluation and in sonography scores in diet group. No statistically significant changes in CDAI, IBD-questionnaire, or inflammatory parameters. |
| Charlebois | 2015 | 1 | CD | Remission | Semi-Vegetarian Diet vs. omnivorous diet | Significantly higher clinical remission rates. |
| Charlebois | 2015 | 1 | UC | Progression | Salmon rich diet (600 g of salmon/week) | Significant improvements in SCCAI-score (simple CD/UC activity index), and nonsignificant reductions in CRP and homocysteine levels. |
| Wedlake | 2014 | 23 | IBD | Progression | Fiber | No evidence for restricting fiber in IBD patents, excluding GI-obstructive complications |
| Wedlake | 2014 | 10 | UC | Progression | Fiber | There is limited weak evidence for the efficacy of fiber in improving disease outcomes in UC, more research is needed. |
| Wedlake | 2014 | 12 | CD | Progression | Fiber | Studies reported showed no effect, more research is needed |
| Langhorst | 2014 | 2 | UC | Progression | Curcumin | One study found significantly less disease activity in curcumin group, other study found no significant difference. |
| Langhorst | 2014 | 1 | UC | Progression | Germinated Barley Foodstuff (GBF) | Associated with decrease of steroid use, clinical symptoms and relapse |
| Spooren | 2013 | 2 | UC | Incidence | Cereals and bread intake | Conflicting or non-significant findings (fiber) |
| Spooren | 2013 | 7 | UC | Incidence | Sugar and sugar-containing foods | Increased intake of sugar or sugar-containing food increase risk of UC |
| Spooren | 2013 | 5 | UC | Incidence | Fruits and vegetables | Significant decreased risk of UC with a higher intake of fruits and vegetables. |
| Spooren | 2013 | 8 | CD | Incidence | Cereals and bread intake | Increased risk with white bread, decreased with whole grain/meal bread and cereals/muesli. |
| Spooren | 2013 | 12 | CD | Incidence | Sugar and sugar-containing foods | Increased intake of sugar give increase risk of CD. |
| Spooren | 2013 | 10 | CD | Incidence | Fruits and vegetables | Higher intake was associated with a decreased risk, especially citrus fruit. |
| Spooren | 2013 | 1 | UC | Progression | Meat and meat products | Increased risk of relapse |
| Spooren | 2013 | 1 | UC | Progression | Alcohol | Increased risk of relapse |
| Hou | 2011 |  | IBD | Incidence | PUFA | Associated with higher incidence of CD and UC. |
| Hou | 2011 |  | IBD | Incidence | n-6 PUFA | Associated with higher incidence of CD and UC. |
| Hou | 2011 |  | IBD | Incidence | Meat and meat products | Associated with higher incidence of CD and UC. |
| Hou | 2011 |  | CD | Incidence | Fruit | Associated with lower incidence of CD |
| Hou | 2011 |  | CD | Incidence | Fiber | Associated with lower incidence of CD |
| Hou | 2011 |  | UC | Incidence | Vegetables | Associated with lower incidence of UC |

**Supplementary Table S3:** Summary of biomarkers for disease listed by first author and exposure.

| **First author** | **Year** | **Studies (n)** | **Disease** | **Specific** | **Exposure** | **Findings** |
| --- | --- | --- | --- | --- | --- | --- |
| Barros | 2021 | 3 | IBD | Calprotectin | Low-FODMAP diet | No significant reduction in fecal calprotectin, but initial improvement of GI-symptoms |
| Barros | 2021 | 3 | IBD | CRP | Low-FODMAP diet | No significant reduction in CRP, but initial improvement of GI-symptoms |
| Barros | 2021 | 2 | IBD | Calprotectin | IgG-diet | No significant reduction in fecal calprotectin, but a subgroup with CDAI <150 was showing a significant reduction in fecal calprotectin in patients on the true diet compared to the control one |
| Barros | 2021 | 2 | IBD | CRP | IgG-diet | No significant reduction in CRP |
| Barros | 2021 | 1 | CD | Calprotectin | diet to reduce food microparticles (titanium dioxide and silicate) | No significant reduction in fecal calprotectin |
| Barros | 2021 | 1 | CD | CRP | diet to reduce food microparticles (titanium dioxide and silicate) | No significant reduction in CRP |
| Barros | 2021 | 1 | CD | CRP | Semi-vegetarian diet | Significant reduction in CRP (also 92% 2 year remission rate compared to omnivorus diet) |
| Barros | 2021 | 1 | IBD | Calprotectin | autoimmune protocol (AIP) diet (elimination of some specific foods (grains, vegetables, eggs, milk, coffee, alcohol, nuts and seeds, refined sugar, additives, and processed foods) for six weeks) | Improvement in the endoscopic score of patients with CD and a significant reduction in fecal calprotectin |
| Barros | 2021 | 1 | IBD | CRP | autoimmune protocol (AIP) diet (elimination of some specific foods (grains, vegetables, eggs, milk, coffee, alcohol, nuts and seeds, refined sugar, additives, and processed foods) for six weeks) | No significant change in CRP values, but improvement in the endoscopic score of patients with CD |
| Barros | 2021 | 1 | UC | CRP | olive oil vs canola oil | Improvement in gastrointestinal symptoms for UC and significant reduction in CRP and ERS, without changes in TNF-α concentrations, while the group that consumed canola oil did not obtain significant changes in these inflammatory biomarkers |
| Barros | 2021 | 1 | IBD | CRP | hydrothermally processed cereals (active food) | Levels of active foods in the active group were significantly higher than in the placebo group, but there were no significant differences between groups for CRP |
| Barros | 2021 | 1 | IBD | anti-secretory factor (AF) | hydrothermally processed cereals (active food) | Levels of active foods in the active group were significantly higher than in the placebo group |
| Barros | 2021 | 1 | IBD | CRP | germinated barley | No significant difference in CRP between the control diet and germinated barley groups |
| Barros | 2021 | 1 | UC | CRP | Atlantic salmon | No significant reductions in CRP in patients with UC |
| Barros | 2021 | 1 | UC | TNF- α | Atlantic salmon | No significant changes in TNF- α in patients with UC |
| Barros | 2021 | 1 | IBD | pro-inflammatory cytokines | Mango | Reduction in pro-inflammatory cytokines |
| Barros | 2021 |  | IBD | CRP | increasing the intake of vitamin B6 and folate | No change in homocysteine levels, but a significant decrease in serum concentrations of CRP |
| Barros | 2021 |  | CD | CRP | More fiber and less refined carbohydrates | Clinical improvement of the disease, but no significant differences in CRP and ESR between the test and control groups in the four weeks of the study |
| SaeidiFard | 2020 | 1 | IBD | CRP | Fermented foods (yogurt) | MD: 4300.00 (3556.62, 5043.38) |
| SaeidiFard | 2020 | 1 | IBD | IL-6 | Fermented foods (yogurt) | MD: 18.00 (17.16, 18.84) |
| SaeidiFard | 2020 | 1 | IBD | TNF-alpha | Fermented foods (yogurt) | MD: -93.00 (-94.42, -91.58) |
| Montroy | 2020 | 4 | IBD | Short chain fatty acid concentration | Resistant starches (RS) | Significant increase in short chain fatty acid concentration (3/4 studies), while no difference in concentration but an increase in butyrate alone (1/4 studies) |
| Montroy | 2020 | 2 | IBD | Stool consistency and/or frequency | Resistant starches (RS) | High intake of resistant starches and wheat bran was associated with a significantly shorter whole gut transit time i healthy control patients, but no significant difference was seen in UC-patients. Significant correlation between the proportion of resistant starches and the solids recovered in effluent. |
| Montroy | 2020 | 1 | IBD | CRP | Germinated Barley Foodstuff (GBF) | No statistically significant change, however authors suggested there was a trend towards decreasing levels |
| Peng | 2022 | 3 | IBD | Fecal calprotectin | Low-FODMAP diet | MD -16.03 (-36.78, 4.73) |
| Comeche | 2021 |  | IBD | biomarkers | Low-FODMAP diet | Some authors found improvement i biomarkers, other did not |
| Comeche | 2021 |  | IBD | CRP | Mediterranean diet | Some authors reported significant reduction, others did not |
| Comeche | 2021 |  | IBD | CRP | The Immunoglobulin Exclusion Diet (IGED) | Contradictory results |
| Comeche | 2021 |  | IBD | CRP, fibrinogen, total leukocytes | Vegetarian diet | Associated with decrease in CRP, fibrinogen and total leukocytes |

**Supplementary Table S4:** Summary of quality grading listed by first author and AMSTAR-2 item number with total grading.

| First author | Item1 | Item2 | Item3 | Item4 | Item5 | Item6 | Item7 | Item8 | Item9 | Item10 | Item11 | Item12 | Item13 | Item14 | Item15 | Item16 | AMSTAR-2 |
| --- | --- | --- | --- | --- | --- | --- | --- | --- | --- | --- | --- | --- | --- | --- | --- | --- | --- |
| Barros | Yes | Yes | Yes | Yes | Yes | Yes | Yes | Yes | Yes | No | NR | NR | Yes | NR | NR | Yes | High |
| Charlebois | Yes | Partly | Yes | Yes | Yes | No | Yes | Yes | No | No | NR | NR | No | NR | NR | No | Critically low |
| Coelho | Yes | Yes | Yes | Yes | Yes | Yes | Yes | Yes | Yes | No | NR | NR | Yes | NR | NR | Yes | High |
| Comeche | Yes | Partly | Yes | Yes | Yes | No | Yes | Yes | Yes | No | Yes | Yes | Yes | Yes | Yes | Yes | High |
| Cooper | Yes | No | Yes | Yes | Yes | Yes | Yes | Yes | Yes | No | NR | NR | No | NR | NR | Yes | Low |
| Franqui | No | No | Yes | Yes | No | No | Yes | No | No | No | NR | NR | No | NR | NR | Yes | Critically low |
| Ge | Yes | Partly | Yes | Yes | Yes | No | Yes | Yes | Yes | Yes | Yes | Yes | Yes | Yes | Yes | Yes | High |
| Güngör | Yes | Partly | Yes | Yes | Yes | Yes | Yes | Yes | Yes | Yes | NR | NR | Yes | NR | NR | Yes | High |
| Irrazabal | No | No | No | No | No | No | No | Yes | No | No | NR | NR | No | NR | NR | Yes | Critically low |
| Jaber | Yes | Yes | Yes | Yes | Yes | No | Yes | Yes | Yes | No | NR | NR | No | NR | NR | Yes | High |
| Jin | Yes | Yes | Yes | Yes | Yes | Yes | Yes | Yes | Yes | No | Yes | Yes | Yes | Yes | Yes | No | High |
| Khademi | Yes | Partly | Yes | Yes | No | Yes | Yes | Yes | Yes | No | Yes | Yes | Yes | Yes | No | Yes | Moderate |
| Khorshidi | Yes | No | Yes | Yes | No | Yes | Yes | Yes | Yes | No | Yes | Yes | Yes | Yes | Yes | Yes | Low |
| Langhorst | Yes | Partly | Yes | Yes | Yes | Yes | Yes | Yes | Yes | No | NR | NR | Yes | NR | NR | Yes | High |
| Li | Yes | Partly | Yes | Yes | Yes | Yes | Yes | Yes | Yes | No | Yes | Yes | Yes | Yes | Yes | Yes | High |
| Li | Yes | Partly | Yes | Yes | Yes | Yes | Yes | Yes | No | No | Yes | Yes | Yes | Yes | Yes | Yes | Low |
| Limketkai | Yes | No | Yes | Yes | Yes | Yes | Yes | Yes | Yes | Partly | Yes | Yes | Yes | Yes | No | Yes | Low |
| Limketkai | Yes | Yes | Yes | Yes | Yes | Yes | Yes | Yes | Yes | Yes | NR | NR | Yes | NR | NR | Yes | High |
| Liu | Yes | Partly | Yes | Yes | Yes | Yes | Yes | Yes | Yes | No | Yes | Yes | Yes | Yes | Yes | Yes | High |
| Marsh | Yes | Yes | Yes | Yes | Yes | Yes | Yes | Yes | Yes | No | NR | NR | Yes | NR | NR | Yes | High |
| McCormick | Yes | Partly | Yes | Yes | No | No | Yes | Yes | Yes | No | NR | NR | Yes | NR | NR | Yes | Moderate |
| Milajerdi | Yes | Partly | Yes | Yes | No | Yes | Yes | Yes | Yes | No | Yes | Yes | Yes | No | No | Yes | Critically low |
| Montroy | Yes | Yes | Yes | Yes | Yes | Yes | Yes | Yes | Yes | Yes | NR | NR | Yes | NR | NR | Yes | High |
| Mozaffari | Yes | Yes | Yes | Yes | Yes | Yes | Yes | Yes | Yes | Yes | Yes | Yes | Yes | Yes | Yes | Yes | High |
| Narula | Yes | Yes | Yes | Yes | Yes | Yes | Yes | Yes | Yes | No | Yes | Yes | Yes | Yes | Yes | Yes | High |
| Nie | Yes | Partly | Yes | Yes | Yes | No | Yes | Yes | Yes | No | Yes | Yes | Yes | Yes | Yes | Yes | High |
| Penagini | Yes | Partly | Yes | Yes | No | No | Yes | Yes | No | No | NR | NR | No | NR | NR | Yes | Critically low |
| Peng | Yes | Yes | Yes | Yes | Yes | Yes | Yes | Yes | Yes | No | Yes | Yes | Yes | Yes | Yes | Yes | High |
| Peters | Yes | Partly | Yes | No | Yes | No | Yes | Yes | Yes | No | NR | NR | Yes | NR | NR | Yes | Low |
| Rocha | Yes | Partly | Yes | Yes | Yes | No | Yes | Yes | No | No | NR | NR | No | NR | NR | Yes | Low |
| SaeidiFard | Yes | Partly | Yes | Yes | Yes | Yes | Yes | Yes | Yes | No | Yes | Yes | Yes | Yes | Yes | Yes | High |
| Shahinfar | Yes | Partly | Yes | Yes | Yes | Yes | Yes | Yes | Yes | No | Yes | Yes | No | Yes | Yes | Yes | High |
| Sinopoulou | Yes | Yes | Yes | Yes | Yes | Yes | Yes | Yes | Yes | Yes | Yes | Yes | Yes | Yes | Yes | Yes | High |
| Sinopoulou | Yes | Yes | Yes | Yes | Yes | Yes | Yes | Yes | Yes | Yes | Yes | Yes | Yes | Yes | Yes | Yes | High |
| Spooren | Yes | Partly | Yes | Yes | Yes | No | Yes | Yes | No | Yes | NR | NR | Yes | NR | NR | Yes | Low |
| Szilagyi | Yes | Partly | Yes | Yes | Yes | No | Yes | Yes | Yes | No | Yes | Yes | No | Yes | Yes | Yes | High |
| Talebi | Yes | Yes | Yes | Yes | Yes | Yes | Yes | Yes | Yes | No | Yes | Yes | Yes | Yes | Yes | Yes | High |
| Tian | Yes | Yes | Yes | Yes | No | No | Yes | Yes | Yes | No | NR | NR | Yes | NR | NR | Yes | Moderate |
| Valido | Yes | Yes | Yes | Yes | Yes | Yes | Yes | Yes | Yes | No | NR | NR | Yes | NR | NR | Yes | High |
| Wagenaar | Yes | Yes | Yes | Yes | Yes | Yes | Yes | Yes | Yes | No | NR | NR | Yes | NR | NR | Yes | High |
| Wedlake | Yes | Partly | Yes | Yes | Yes | Yes | Yes | Yes | Yes | No | NR | NR | Yes | NR | NR | Yes | High |
| Wu | Yes | Partly | Yes | Yes | Yes | Yes | Yes | Yes | No | No | Yes | Yes | Yes | Yes | Yes | Yes | Low |
| Zhan | Yes | Partly | Yes | Yes | Yes | Yes | Yes | Yes | Yes | Yes | Yes | Yes | Yes | No | Yes | Yes | Low |
| Zhang | Yes | Partly | Yes | Yes | No | Yes | Yes | Yes | Yes | No | Yes | Yes | Yes | Yes | Yes | Yes | High |
| Zhao | Yes | No | Yes | Yes | Yes | Yes | Yes | Yes | Yes | No | Yes | Yes | Yes | Yes | Yes | Yes | Low |
| Zhou | Yes | Partly | Yes | Yes | Yes | Yes | Yes | Yes | Yes | No | Yes | Yes | Yes | Yes | Yes | Yes | High |

**Supplementary Table S5:** Summary of specific dietary patterns.

| **Diet** | **Emphasis on** | **Restrictions** | **Origin** |
| --- | --- | --- | --- |
| Mediterranean diet | Plant-based foods, whole grains, fruits, vegetables, fish | Limited red and processed meat, and refined sugars | Based on a diet traditionally found around the Mediterranean. |
| Semi-vegetarian diet | Mostly plant-based foods | Limited red meat, processed foods | Flexitarian diet, vegetarian diet. |
| Specific carbohydrate diet (SCD) | Specific carbohydrate restrictions, whole foods | Limits food with disaccharides, most polysaccharides; allows monosaccharides | Based on the hypothesis that IBD patients are not able to digest and absorb disaccharides and amylopectin properly |
| Western diet | Emphasis on processed foods, red meat, refined sugars, often fast foods | Limited with whole foods, high in sugars | American eating trends until recently. |
| Low-FODMAP diet | Balanced meals with low FODMAP ingredients (see restrictions) | Avoids certain fermentable carbohydrates (oligo-, di- and monosaccharides) and polyols. | Based on the hypothesis that FODMAPs cause GI-symptoms with bloating, flatulence and abdominal pain when ingested by FODMAP sensitive people. |

**Supplementary Figure S1:** Summary of associations from the meta-analyses meat and meat products and incidence of inflammatory bowel disease (IBD), ulcerative colitis (UC) and Crohn’s' disease (CD).

* Re-analyzed through studies extracted from Ge et al, Mozaffari et al, Talebi et al, Zhou et al.

**Supplementary Figure S2a:** Summary of associations from the meta-analyses on total meat and incidence of inflammatory bowel disease (IBD).

* Re-analyzed through studies extracted from Ge et al, Mozaffari et al, Talebi et al, Zhou et al.

**Supplementary Figure S2b:** Summary of associations from the meta-analyses on red meat and incidence of inflammatory bowel disease (IBD).

* Re-analyzed through studies extracted from Ge et al, Mozaffari et al, Talebi et al, Zhou et al.

**Supplementary Figure S2c:** Summary of associations from the meta-analyses on processed meat and incidence of inflammatory bowel disease (IBD).

* Re-analyzed through studies extracted from Ge et al, Mozaffari et al, Talebi et al, Zhou et al.

**Supplementary Figure S2d:** Summary of associations from the meta-analyses on white meat and incidence of inflammatory bowel disease (IBD).

* Re-analyzed through studies extracted from Ge et al, Mozaffari et al, Talebi et al, Zhou et al.

**Supplementary Figure S2e:** Summary of associations from the meta-analyses on fish and incidence of inflammatory bowel disease (IBD).

* Re-analyzed through studies extracted from Ge et al, Mozaffari et al, Talebi et al, Zhou et al.

**Supplementary Figure S3a:** Summary of associations from the meta-analyses on total meat and incidence of ulcerative colitis (UC).

* Re-analyzed through studies extracted from Ge et al, Mozaffari et al, Talebi et al, Zhou et al.

**Supplementary Figure S3b:** Summary of associations from the meta-analyses on red and processed meat and incidence of ulcerative colitis (UC).

* Re-analyzed through studies extracted from Ge et al, Mozaffari et al, Talebi et al, Zhou et al.

**Supplementary Figure S3c:** Summary of associations from the meta-analyses on red meat and incidence of ulcerative colitis (UC).

* Re-analyzed through studies extracted from Ge et al, Mozaffari et al, Talebi et al, Zhou et al.

**Supplementary Figure S3d:** Summary of associations from the meta-analyses on processed meat and incidence of ulcerative colitis (UC).

* Re-analyzed through studies extracted from Ge et al, Mozaffari et al, Talebi et al, Zhou et al.

**Supplementary Figure S3e:** Summary of associations from the meta-analyses on white meat and incidence of ulcerative colitis (UC).

* Re-analyzed through studies extracted from Ge et al, Mozaffari et al, Talebi et al, Zhou et al.

**Supplementary Figure S3f:** Summary of associations from the meta-analyses on fish and incidence of ulcerative colitis (UC).

* Re-analyzed through studies extracted from Ge et al, Mozaffari et al, Talebi et al, Zhou et al.

**Supplementary Figure S4a:** Summary of associations from the meta-analyses on total meat and incidence of Crohn’s' disease (CD).

* Re-analyzed through studies extracted from Ge et al, Mozaffari et al, Talebi et al, Zhou et al.

**Supplementary Figure S4b:** Summary of associations from the meta-analyses on red meat and incidence of Crohn’s' disease (CD).

* Re-analyzed through studies extracted from Ge et al, Mozaffari et al, Talebi et al, Zhou et al.

**Supplementary Figure S4c:** Summary of associations from the meta-analyses on processed meat and incidence of Crohn’s' disease (CD).

* Re-analyzed through studies extracted from Ge et al, Mozaffari et al, Talebi et al, Zhou et al.

**Supplementary Figure S4d:** Summary of associations from the meta-analyses on white meat and incidence of Crohn’s' disease (CD).

* Re-analyzed through studies extracted from Ge et al, Mozaffari et al, Talebi et al, Zhou et al.

**Supplementary Figure S4e:** Summary of associations from the meta-analyses on fish and incidence of Crohn’s' disease (CD).

* Re-analyzed through studies extracted from Ge et al, Mozaffari et al, Talebi et al, Zhou et al.

**Supplementary Figure S5:** Summary of associations from the meta-analyses between dietary exposures and relapse/progression of ulcerative colitis. Reference number is listed in brackets and search year is listed within the parentheses.


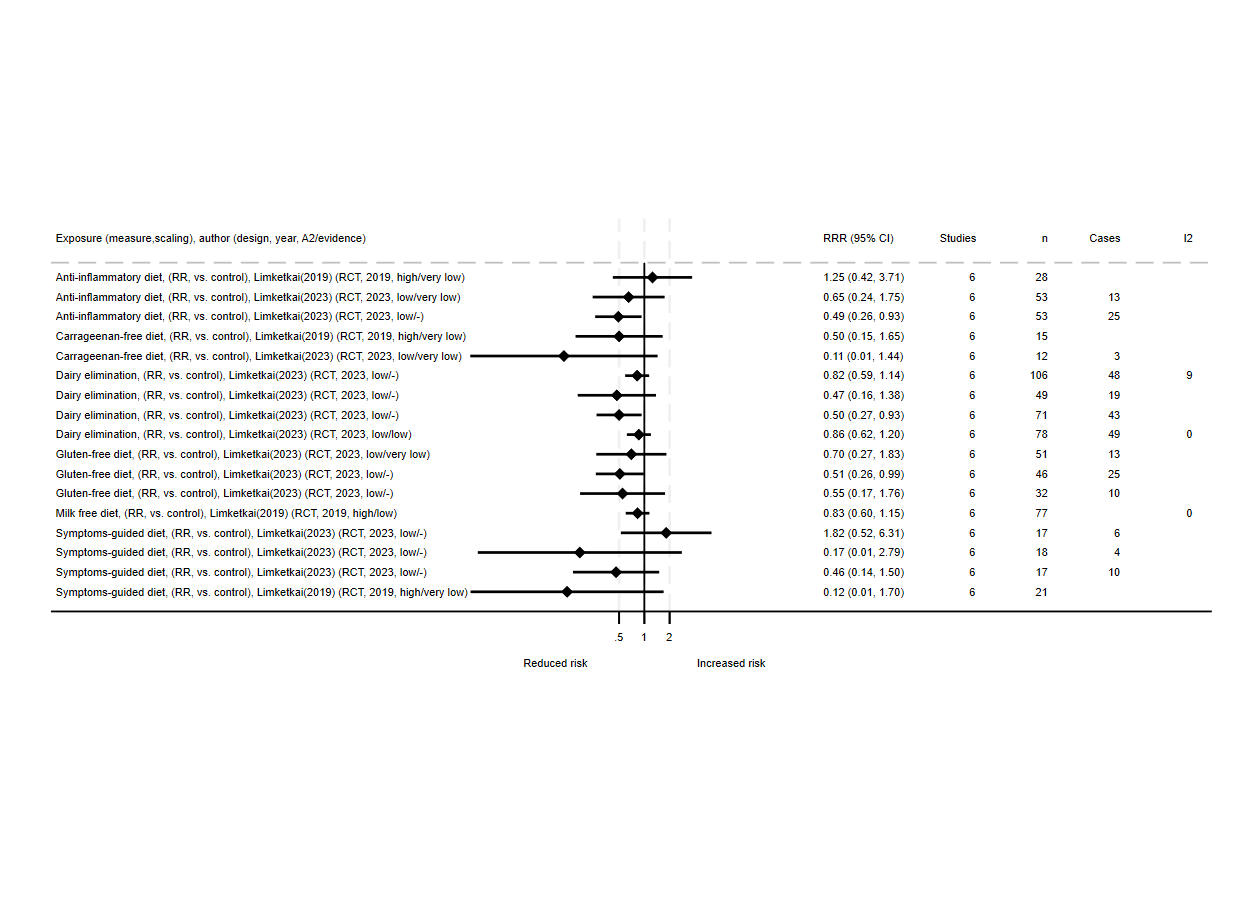


* Comparisons are mostly compared control diets and presented by relative risks (RR). A2: AMSTAR-2 rating (classified as high/medium/low/critically low). RCT: randomized controlled trial; I^2^: heterogeneity (%).

**Supplementary Figure S6:** Summary of associations from the meta-analyses between dietary exposures and relapse/progression of Crohn’s' disease. Reference number is listed in brackets and search year is listed within the parentheses.


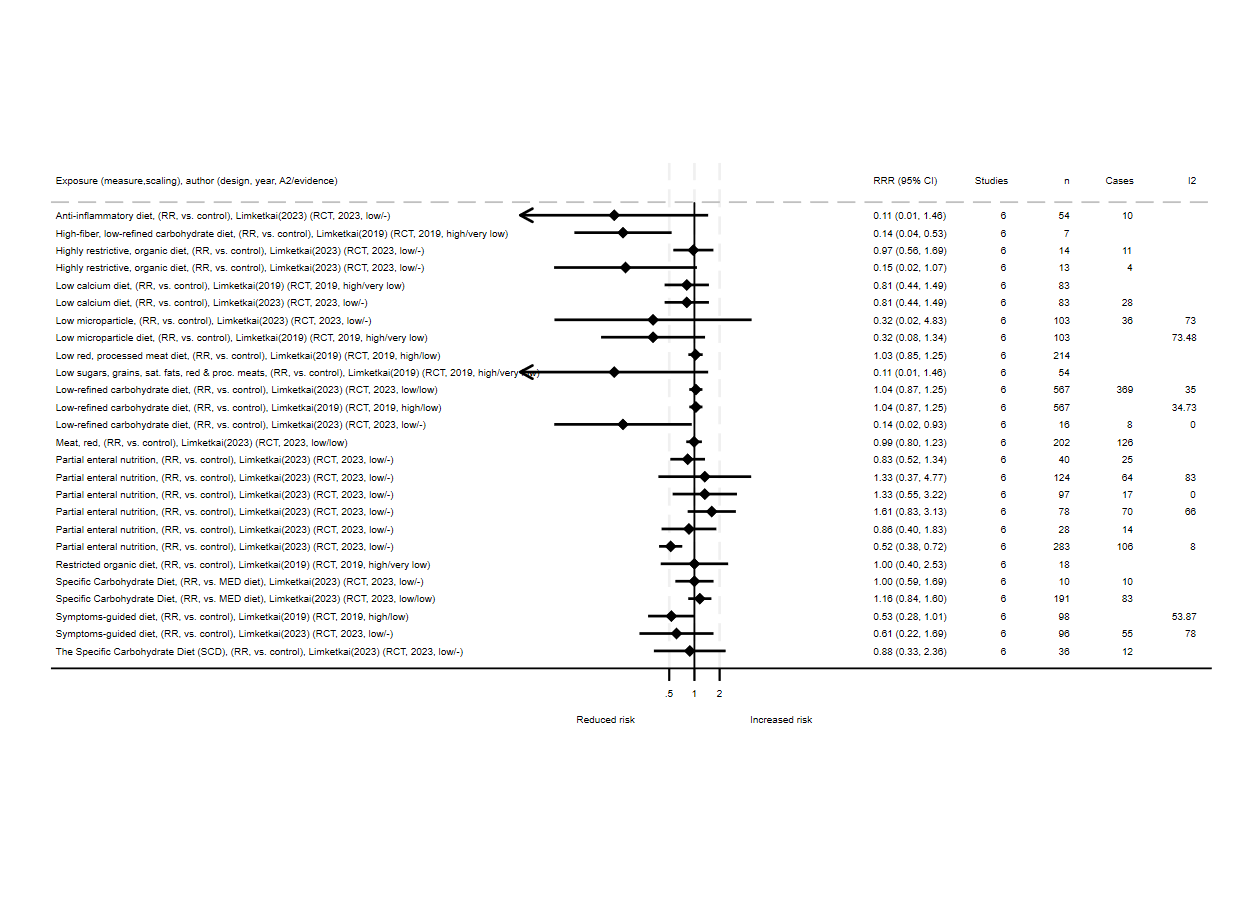


* Comparisons are mostly compared control diets and presented by relative risks (RR). A2: AMSTAR-2 rating (classified as high/medium/low/critically low). RCT: randomized controlled trial; I^2^: heterogeneity (%).

**Supplementary Figure S7:** Summary of associations from the meta-analyses between low-FODMAP diets compared to control diets and symptoms of inflammatory bowel disease. Reference number is listed in brackets and search year is listed within the parentheses.


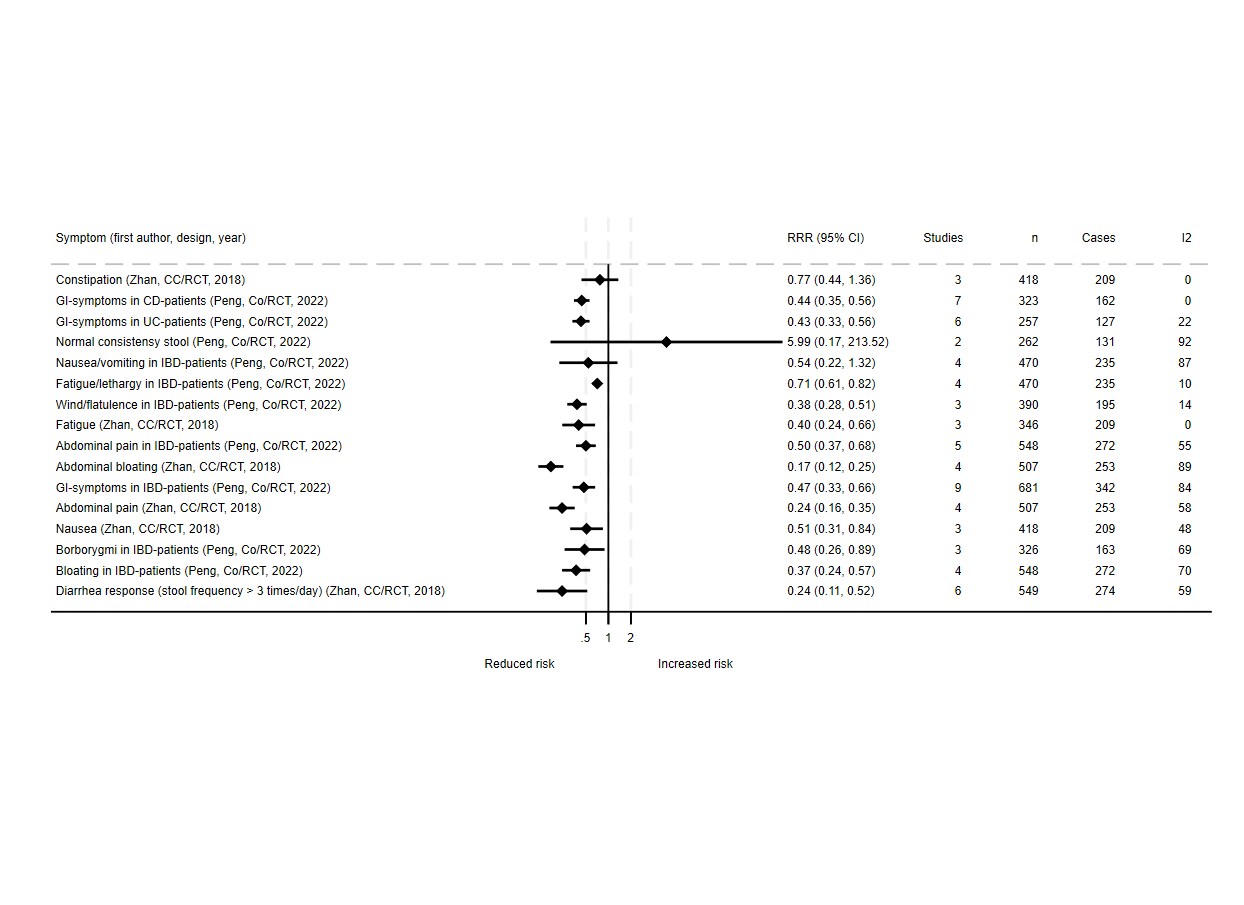


* FODMAPs: fermentable oligosaccharides, disaccharides, monosaccharides, and polyols. Comparisons are presented by relative risks (RR). Co: cohort; CC: case control; RCT: randomized controlled trial; I^2^: heterogeneity (%).
